# Early Deployment of an Integrated Digital Platform (shamiriOS) for Scalable Youth Mental Health Service Delivery in Kenya: Development and Usability Study

**DOI:** 10.2196/79107

**Published:** 2026-06-03

**Authors:** Shadrack Lilan, Tom L Osborn, Wendy Mmbone, Jean Kasudi, Benny Otieno, Ichami Etyang, Jianing Tu, Edmund Korley, Christine Wasanga

**Affiliations:** 1 Shamiri Institute Nairobi Kenya; 2 Columbia University New York, NY United States; 3 The Agency Fund San Francisco, CA United States; 4 Kenyatta University Nairobi Kenya

**Keywords:** digital health platforms, digital mental health, task-shifting, stepped-care, youth mental health, open-source platforms, implementation science, AI in mental health, artificial intelligence, low- and middle-income countries, Kenya

## Abstract

**Background:**

Scaling youth mental health services in low-resource settings requires digital infrastructure that supports not just clinical delivery but the full operational, supervisory, and engagement demands of community-based, task-shifted models. Existing platforms—whether commercial health systems, open-source medical records, or consumer-facing wellness apps—address fragments of this need, but none provide the integrated, offline-capable, and affordable architecture required for lay-provider delivery at scale.

**Objective:**

We introduce shamiriOS, an open-source, modular digital platform comprising 3 interlinked suites—the Shamiri Digital Hub (SDH) for operational management, Rafi for youth engagement, and the Shamiri Provider Platform (SPP) for clinical workflows—designed for scalable, stepped-care youth mental health delivery in Kenya. Our objectives were (1) to conduct an environmental scan of existing platforms and characterize their limitations, (2) to describe the user-centered design and development of shamiriOS, and (3) to report early deployment outcomes across centralized and decentralized settings between 2023 and 2024.

**Methods:**

We conducted a structured environmental scan of 6 case management platforms and 28 youth-facing mental health apps, assessing cost, usability, open-source availability, customizability, offline capability, and suitability for task-shifted delivery. Based on identified gaps, SDH was built as a browser-based operation platform, and Rafi was developed as a native mobile app (Android or iOS) with an offline-first architecture. The SPP was adapted from an existing electronic medical record system. Development followed a user-centered design process with community consultation, including cocreation workshops with 77 university-aged youths. Deployment was evaluated using use analytics, usability ratings, and Net Promoter Scores.

**Results:**

No reviewed platform met the combined requirements for stepped-care delivery. SDH was deployed across 11 sites serving 76,344 youths via 1195 lay providers and 111 clinical supervisors by Q1 2024. Staff reported high satisfaction (usability: mean 8.36/10, SD 1.49; Net Promoter Score: mean 8.63/10, SD 1.46). Rafi achieved 74.7% (n=3737) registration at Mount Kenya University, with 50.4% (508/1008) booking therapy sessions, but sustained engagement with self-guided features declined to near 0 by 9 months.

**Conclusions:**

shamiriOS demonstrates the feasibility of building modular, open-source digital infrastructure for scalable, task-shifted youth mental health delivery. Its component-based architecture is designed for adaptation to other contexts, though extension would require participatory redesign. The most significant obstacles to impact lie not in platform design but in implementation readiness, incentive alignment, and institutional integration. Future priorities include SPP deployment, artificial intelligence–assisted supervision features (shamiriAI), and strengthening sustained engagement.

## Introduction

### Background

Youth mental health challenges are among the most pressing global health issues today [1,2]. Half of all lifetime mental disorders begin by age 14 [3,4]. Most go untreated—particularly in low- and middle-income countries (LMICs), where shortages of trained clinicians and fragile health systems produce a significant treatment gap [1,2,5,6]. Closing this gap requires care models that are cost-effective at scale, contextually grounded, and delivered beyond traditional clinical settings into community spaces [1,7,8].

### Stepped Care Models Can Close the Treatment Gap

Task-shifting—training nonprofessionals to deliver structured, evidence-based treatments under supervision—has emerged as a promising strategy for closing the treatment gap [9,10]. When embedded within stepped-care systems, where the intensity of care is matched to individual need [9-12], this approach maximizes scarce resources, reduces costs, and leverages community infrastructure for delivery [12,13].

**Figure 1:**
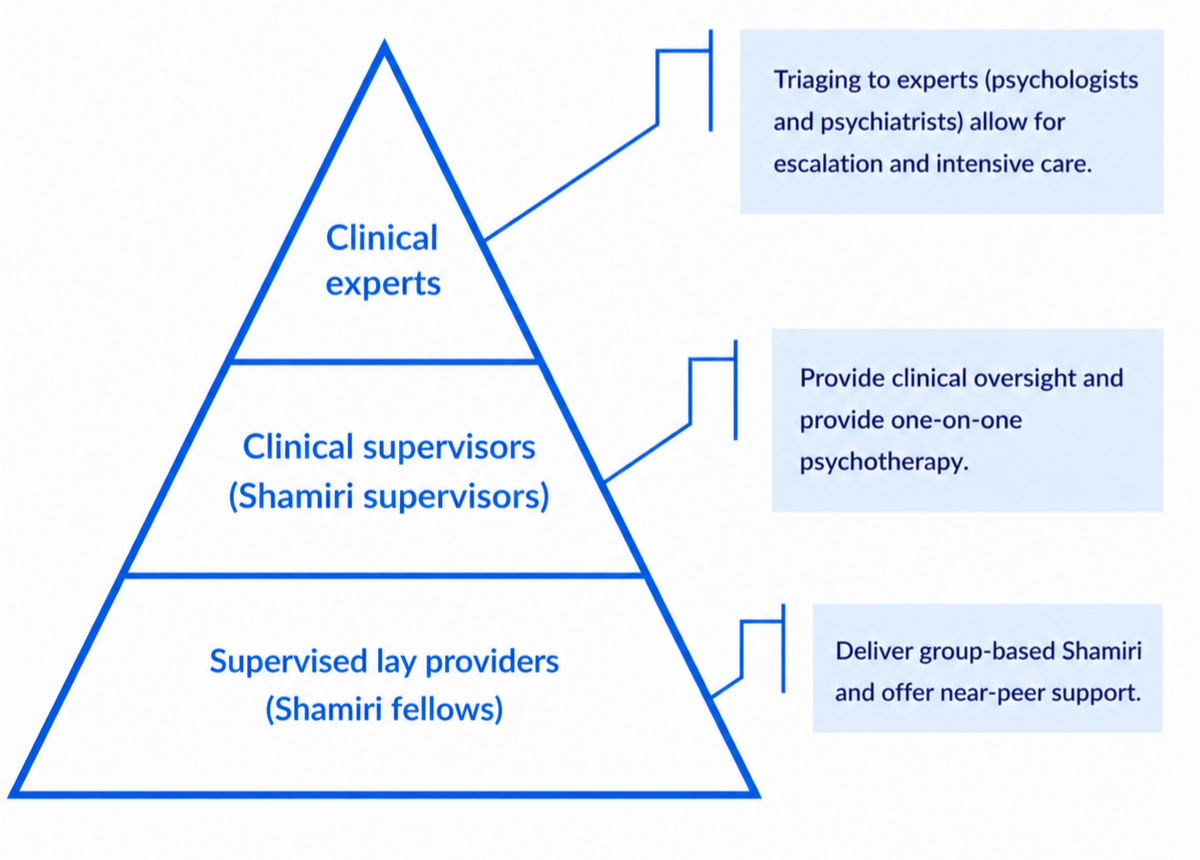
The Shamiri model: a 3-tier stepped care model for youth mental health.

The Shamiri model is an example of task-shifting within a stepped-care system: a 3-tier school-based mental health program delivered by lay providers and clinical staff (Figure 1) [14,15]. In the first tier, lay providers aged 18-22 years are trained and supervised to deliver brief, group-based psychological interventions—typically weekly sessions of 4 weeks with groups of 6 to 15 students [16,17]. Clinical supervisors with backgrounds in psychology or clinical social work form the second tier, providing oversight of lay providers and direct one-on-one psychotherapy where needed [16,18,19]. Severe or complex cases are escalated to clinical psychologists and psychiatrists in the third tier [16,18,19]. Randomized controlled trials demonstrate that the Shamiri model significantly reduces depression and anxiety symptoms in secondary school students, with effects sustained at least 7 months after the intervention [14,15,20,21]. Cost-effectiveness analyses place delivery at approximately US $7.48 per student in 2024—among the most affordable youth mental health interventions globally [22,23].

Despite these outcomes, scaling innovations like Shamiri while maintaining fidelity and efficacy across diverse contexts remains a challenge. Two main dissemination strategies have been used [24,25]. The centralized approach, where the originating organization retains direct control, allows for tighter quality control but limits reach [23]. The decentralized—or “train-the-trainer”—approach, where other organizations implement independently, enables rapid scale-up but carries risks to fidelity and consistency [24,25]. Recent research comparing both approaches in Kenya found both effective at improving youth outcomes, though decentralized models require more robust support structures and greater funding to maintain effectiveness and fidelity [23].

As implementation moves across contexts and implementers, additional operational challenges arise—particularly for small or resource-constrained community-based organizations [23,26,27]. Coordinating multitiered teams, managing logistics and financial workflows, tracking real-time outcomes, and maintaining consistent training and supervision all pose barriers to broader uptake of otherwise effective interventions [23,26,27].

### Technology as a Catalyst for Scale

Digital platforms can address these bottlenecks. Purpose-built technology can reduce administrative burden, standardize supervision workflows, enable real-time monitoring, and facilitate client transitions across care tiers [13,28,29]. For task-shifted stepped-care models specifically, digital tools can provide lay providers with structured session prompts, automate operational workflows, support triage, and coordinate across roles [13,28-31].

Most existing digital mental health tools fall short of what community-based delivery in Kenya—and similar contexts—requires [13,30-32]. Available platforms are typically designed for high-resource clinical environments, carry prohibitive licensing costs, lack customizability for task-shifted workflows, and offer little or no offline functionality [29,33,34]. Multiple recent reviews confirm this infrastructure gap: digital mental health interventions developed for LMICs remain limited in number, and those that exist rarely include the operational architecture needed for stepped-care delivery, provider supervision, or scalable implementation [29,35-37]. Platforms built to coordinate lay-provider delivery, integrate operational and clinical workflows, and function in bandwidth-constrained settings are largely absent from the literature [29,37]. This gap is not unique to Kenya: across sub-Saharan Africa, South Asia, and Southeast Asia—settings characterized by severe mental health workforce shortages and limited digital infrastructure—the distance between the promise of digital health tools and the availability of platforms suited for community-based delivery remains wide [1,38].

### Development of shamiriOS: A 3-Product Platform for Delivery at Scale

**Figure 2:**
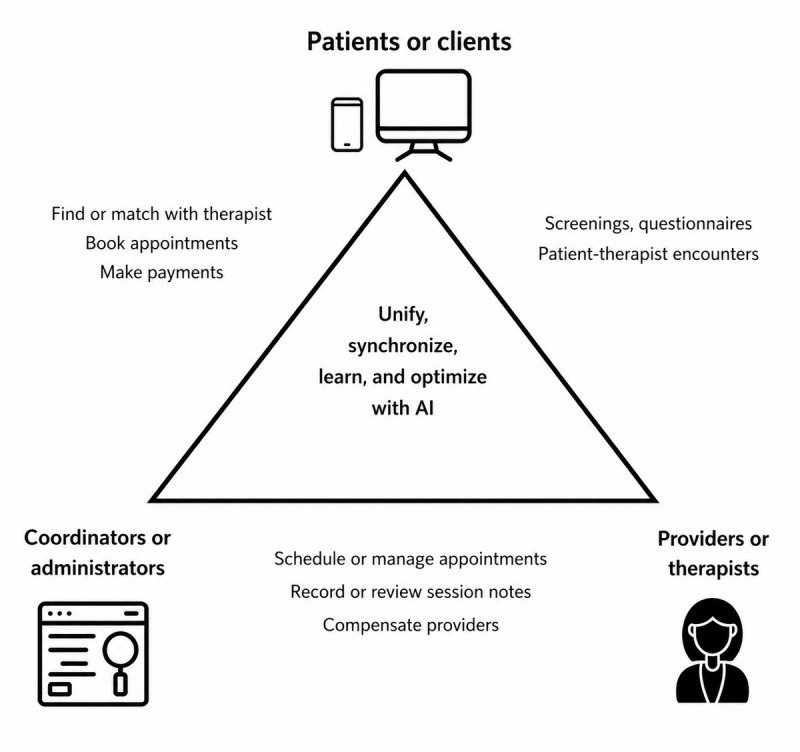
shamiriOS overview. AI: artificial intelligence.

To address this gap, we developed shamiriOS—an open-source digital platform built to support the scalable delivery of task-shifted youth mental health services in Kenya and similar settings. The name reflects its purpose: an integrated operating system for service delivery, not a conventional computer operating system. The platform comprises 3 interoperable product suites—the Shamiri Digital Hub (SDH), the Shamiri Provider Platform (SPP), and Rafi—each designed for a distinct user group within a task-shifted care approach like the Shamiri model (Figure 2):

- SDH is an operational command center for program management, coordination, supervision oversight, and financial workflows.
- SPP is a clinical workflow tool supporting fellows, supervisors, and therapists in delivering and documenting care.
- Rafi is a client-facing mobile app through which young people access care, navigate support pathways, and engage with mental health resources. Available on Android and iOS, Rafi is built with an offline-first architecture to remain functional in the low-bandwidth environments common across Kenyan schools and universities.

These 3 products function as a unified delivery ecosystem: SDH runs the program, SPP supports the providers, and Rafi connects young people to care. Each component can be deployed independently or in combination, depending on an implementing organization’s context and capacity. Additionally, each component is “modular” in design: its features can be configured and adapted to meet the specific needs of different implementing partners, contexts, and end users.

Although the broader vision for shamiriOS encompasses all 3 components, this paper focuses primarily on the development and deployment of SDH and Rafi—built from the ground up—and on the initial integration of SPP, adapted from an existing clinical platform. shamiriOS builds on the capabilities and limitations of existing platforms, including case management systems such as CommCare and OpenMRS (Open Medical Record System), and youth-facing tools reviewed in our environmental scan [29,35-37]. Its distinctive contribution is an integrated, 3-product architecture combining operational management, clinical workflows, and client-facing engagement within a single open-source, context-specific system—a combination not found in existing platforms in Kenya or comparable settings. Successful adaptation for other countries is feasible with appropriate participatory redesign; the findings reported here are specific to the Kenyan context in which the platform was developed and validated.

### Study Aims

This paper has 3 aims. First, we present an environmental scan of existing digital platforms to characterize their limitations for stepped-care youth mental health delivery in Kenya. Second, we describe the iterative, user-centered design and development process of shamiriOS. Third, we evaluate early outcomes from its deployment across centralized and decentralized implementation settings in Kenya between 2023 and 2024, reporting on adoption, user satisfaction, and feature refinement.

Together, these 3 elements—environmental scan, platform development, and early deployment—establish a foundational evidence layer for the ongoing development, evaluation, and contextual adaptation of integrated digital mental health platforms in settings where such infrastructure is critically needed.

## Methods

### Overview

This section describes the review, development, and deployment processes underlying shamiriOS. We first describe the environmental scan of existing platforms that informed our decision to build a custom solution. We then outline the iterative development of each component—the SDH and Rafi, built from the ground up, and the SPP, adapted from an existing clinical system. We describe technical access specifications, the engagement measurement framework used to evaluate deployment outcomes, and the phased deployment of the platform across centralized and decentralized settings in Kenya between 2023 and 2024.

### Environmental Scan of Existing Platforms

#### Overview and Evaluation Criteria

Prior to development, we conducted an environmental scan to assess whether existing digital platforms could support the operational, clinical, and engagement requirements of the Shamiri stepped-care model in Kenya. The scan assessed market availability, feature coverage, and contextual fit for community-based, task-shifted delivery—it was not a systematic review of clinical efficacy. We evaluated potential tools across 3 envisioned system functions: a backend operations platform (SDH), a client-facing engagement tool (Rafi), and a clinical provider platform (SPP).

Platforms were evaluated against 6 criteria tailored to the Kenyan implementation context: cost (upfront and ongoing), ease of use for administrators and end users, customizability for task-shifted workflows, open-source availability, suitability for mental health interventions, and appropriateness for low-resource settings including offline capability. These criteria were determined through prior field experience and consultation with Shamiri’s operations and clinical teams (Table 1).

**Table 1:** Platform evaluation criteria and justification.

Criterion	Rationale for LMIC^a^ or Kenyan context
Cost (upfront and ongoing)	Must be affordable for grassroots and mid-sized organizations; includes licensing, hosting, and support costs.
Ease of use	Must be usable by lay providers, supervisors, and adolescent clients with varying levels of digital literacy.
Customizability	Must accommodate task-shifting workflows, school-based implementation, and stepped-care triage.
Open-source availability	Ensures accessibility, adaptability, and long-term sustainability without vendor lock-in.
Suitability for mental health	Requires support for psychosocial content, triage workflows, and group or individual care formats.
Low-resource appropriateness	Must support offline use, low bandwidth environments, and options for local hosting.

^a^LMIC: low- and middle-income country.

#### Backend Operations Platforms

Between May and July 2023, we conducted a desktop scan of global case management platforms through public documentation, product demonstrations, and technical repositories. Six platforms were reviewed: Salesforce, Dimagi CommCare, KoboToolbox, Ona, ODK Collect, and Google Workspace. Platforms were assessed for their ability to support real-time multisite coordination, stipend disbursement tracking, session attendance logging, triage escalation, and role-differentiated access across lay providers, supervisors, and coordinators. Search methods and assessment criteria are described in Multimedia Appendix 1.

#### Client-Facing Youth Mental Health Apps

Searches were conducted on the Google Play Store and Apple App Store using terms including mental health, therapy, depression, and youth wellbeing. Consistent with prior research methodology, we examined the top 10 results for each term [39]. Eligible apps had a minimum of 100,000 downloads (Android) or a 4.0+ star rating (iOS), were available in English and accessible in East Africa, and had been updated within the prior 12 months. Apps focused solely on physical wellness or designed exclusively for clinicians were excluded. A total of 28 apps met eligibility criteria and were assessed across cost structure, provider matching and support features, validated assessment tools, delivery model, self-help resources, and user engagement mechanisms including gamification. Reviewed apps were disaggregated by whether they claim an explicit evidence base (eg, cognitive behavioral therapy and interpersonal therapy) versus general wellness positioning to enable a more precise assessment of their suitability for stepped-care delivery. Full details are in Multimedia Appendix 1.

#### Clinical Provider Platforms

Existing electronic medical record (EMR) and clinical workflow systems were reviewed for suitability as a provider-facing platform, including District Health Information System 2 (DHIS2), OpenMRS, Bahmni, and EasyClinic. Systems were assessed for clinical documentation capability, teletherapy support, appointment scheduling, analytics dashboards, data security, and ease of customization for a task-shifted delivery context.

### Development of the shamiriOS Platform

#### Overview and Design Approach

The environmental scan confirmed that no existing solution fully met the operational, clinical, and youth engagement needs of the Shamiri model in Kenya. We initiated development of shamiriOS in mid-2023, conceptualizing it as a 3-product digital ecosystem: the SDH, Rafi, and the SPP. Initial development focused on building SDH and Rafi from the ground up while adapting SPP from an existing solution, EasyClinic (Table 2).

**Table 2:** Development of shamiriOS components.

Component and version			Period		Features		Feedback
**Shamiri Digital Hub**							
	Version 1	Late 2022 to early 2023		Session attendance tracking, fellow performance monitoring, supervisory reviews		Improved mobile responsiveness, integration of payment and stipend tracking tools, support for multisite coordination	
	Version 2	Early to late 2023		Supervisor and coordinator dashboards, stipend reconciliation workflows, referral tracking, automated task reminders		Iterative feedback via field interviews and monthly user experience reviews	
	Version 3	Late 2023 to present		Multirole platform, multisite implementation management, Metabase-powered analytics dashboards, offline functionality		Development continues, phased rollouts planned for 2025 and beyond	
**Rafi**							
	Version 1.0	May 2022		Daily motivational content, well-being check-ins, therapy and group session booking, monthly score tracking, gamified system		Need for more frequent interaction, personalization, engaging visuals	
	Version 2.0	Early 2023		Daily mood check-ins, interactive reward system, artificial intelligence–generated mood art, triage pathway, community feed		Strong acceptability and usability, need for more modular content, deeper personalization	
	Version 3.0	Early 2024		Expanded self-help toolkit, upgraded triage flows, redesigned interface, anonymous messaging, session scheduling, offline functionality		Feasibility of integrating technology-driven triage and engagement tools into institutional frameworks	
**Shamiri Provider Platform**							
	Version 1.0	2024 to present		Client management, teletherapy support, session documentation, analytics dashboards, syncing user records, real-time therapist availability, appointment scheduling		Emphasis on data security, user-centered design, interoperability	

The development of shamiriOS was guided by a user-centered design process with community consultation, adapted to the operational demands of building integrated health technology infrastructure in Kenya [40,41]. There is no single template for co-design and user-centered design; rather, the methods must be tailored to the nature of what is being designed, the stakeholders involved, and the constraints of the implementation environment [42-45]. The shared philosophy—centering the needs, experiences, and contexts of end users—remained constant across components; the specific methods varied by component type.

For shamiriOS, we distinguish 2 types of design work. The operational infrastructure components—SDH and SPP—were designed primarily as administrative and clinical workflow tools, and their development was guided by consultations with Shamiri’s operations, clinical, and research teams. Rafi, the youth-facing component, was developed through a more participatory co-design process: youth end users shaped interface design, content tone, and feature prioritization through structured feedback sessions, card-sorting exercises, and prototype walkthroughs. Cocreation sessions for the initial version involved 77 students across 3 Kenyan universities (Multimedia Appendix 2).

Across all components, end users—lay providers, clinical supervisors, hub coordinators, and youth participants—acted as consultants and testers, informing design through structured feedback, usability tests, and pain-point mapping. Strategic decisions regarding platform architecture, feature prioritization, and technical implementation remained with Shamiri’s technical and clinical leadership. This approach sits at the consultation end of the community engagement spectrum rather than representing fully community-led development; its implications and our future commitments are discussed in the Discussion section.

#### Development of the SDH

##### Overview

The SDH was designed to replace fragmented operational tools—spreadsheets, WhatsApp messaging, and paper logs—that were prone to error and coordination breakdowns as the number of supervisors, fellows, and implementation sites grew. The goal was a centralized platform enabling real-time monitoring, stipend disbursement, and data-driven supervision across multisite teams.

SDH development occurred across 3 cycles between 2022 and 2024, each guided by user consultation with Shamiri’s operations, clinical, and research teams. Each development cycle followed a hybrid Agile-Waterfall methodology. Agile development is an iterative approach in which work is broken into short, time-boxed cycles called sprints—typically 2 to 4 weeks—with working software produced at the end of each cycle based on user feedback [46]. Waterfall is a sequential approach in which requirements are fully scoped before development begins [47]. The hybrid model we used scoped core feature requirements in advance (Waterfall) but delivered them through sprint-based cycles with regular stakeholder check-ins and usability testing (Agile), allowing responsiveness to evolving user needs while maintaining predictable development milestones [46,47]. This approach maintained predictable milestones while remaining responsive to evolving user needs.

##### Version 1: Prototype Development (Late 2022 to Early 2023)

Version 1 was built on Bubble, a no-code development platform, and served as a minimum viable product to address the most critical operational needs: session attendance tracking, fellow oversight, and performance review. It was deployed in a small usability pilot across 3 implementation hubs and 20 field staff to assess basic usability, workflow alignment, and feasibility of replacing paper-based tracking. User feedback identified 3 priorities for subsequent development: improved mobile responsiveness, integration of payment and stipend tracking, and support for multisite coordination.

##### Version 2: Supervisor-Centered Platform Expansion (Early to Late 2023)

Version 2 rebuilt SDH on a scalable web app architecture: a Node.js and Express backend, a Next.js frontend, and a PostgreSQL database, with components communicating via RESTful (Representational State Transfer) application programming interfaces (APIs), JSON Web Token–based authentication, and role-based access control (RBAC) at the API layer. Full technical specifications, including the data architecture and security model, are in Multimedia Appendix 3.

Version 2 introduced supervisor and coordinator dashboards, stipend reconciliation workflows, referral tracking, and automated task reminders. It was deployed across 5 hubs, with iterative feedback collected via field interviews and monthly user experience reviews.

##### Version 3: Multirole Platform and Offline Functionality (Late 2023 to Present)

Version 3 began development in late 2023 to accommodate scaling demands and a more diverse user base. The platform was expanded to include fellows, supervisors, coordinators, and clinical leads; introduced multisite implementation management; and integrated Metabase-powered analytics dashboards. Metabase is an open-source business intelligence tool that enables nontechnical users to query databases and generate visual reports without writing code [48,49]; its integration allows hub coordinators and supervisors to access real-time program data without developer support. Version 3 also introduced offline-first data capture with automatic synchronization upon network reconnection, supporting use in the low-connectivity environments common across Kenyan schools and community centers. Local server deployment options are under active development.

#### Development of Rafi

##### Overview

Rafi (derived from rafiki, Kiswahili for “friend”) is a client-facing native mobile app for young people—primarily university students aged 17 to 22 years—supporting mental health screenings, patient-therapist encounters, and community engagement within Shamiri’s stepped-care model. Rafi is available on Android (Google Play Store) and iOS (Apple App Store) and was designed with an offline-first architecture: core features—including self-guided content (affirmations, journaling, and goal tracking), mood check-ins, and well-being score tracking—remain functional without internet access. Features requiring connectivity—therapy booking, artificial intelligence (AI)–generated mood artwork, community feed posts, and data synchronization—queue locally and sync automatically on reconnection. This design decision was informed by the environmental scan finding that only 5 of 28 reviewed youth-facing apps offered any offline capability, despite the intermittent-connectivity environments common across Kenyan universities and schools.

##### Conception and Design

Rafi’s development followed the user-centered design process described earlier. Youth end users were engaged as testers and consultants across each version through structured feedback sessions, card-sorting exercises, prototype walkthroughs, and focus group discussions. Cocreation sessions for the initial version involved 77 students across 3 universities (Multimedia Appendix 2). Architectural and design decisions were made by the Shamiri technical team.

##### Rafi 1.0: Initial Prototype

Version 1.0 was developed as a mobile app available on Android, iOS, and a supplementary web portal to maximize accessibility across Kenya’s variable connectivity landscape. Core features included daily and weekly mental health check-ins, access to the Shamiri-Digital self-guided intervention, low-cost caregiver connections, clinical referral pathways, and gamification elements (points and rewards). Version 1.0 was piloted at 7 Kenyan universities through partnerships with peer counseling groups (Multimedia Appendix 2).

##### Rafi 2.0: Stable Platform

Following pilot feedback, version 2.0 introduced mood-based AI-generated artwork (the “wellness gallery”), enhanced triage tools, and expanded the gamification system. It was cocreated with 77 youth users through structured feedback sessions and deployed across select student clubs at additional universities using QR code–based registration and peer ambassador–led outreach.

##### Rafi 3.0: Integrated Platform

Version 3.0 was designed to address persistent retention challenges from earlier deployments. Key design priorities included gamified onboarding sequences, mood-responsive motivational content, and more direct pathways connecting users to therapists. The interface was restructured to reduce feature overload, and the backend was refactored for tighter SPP integration, supporting seamless transitions from self-guided to provider-supported care. Version 3.0 was the platform used in the Mount Kenya University (MKU) institutional deployment described below.

#### Development of the SPP

The SPP was adapted from EasyClinic, an existing EMR solution selected for its modular design, customizable clinical workflows, and prior use in resource-constrained health care settings. Rather than building a clinical platform from the ground up, we adapted an existing, maintained solution to reduce development cost and time to deployment. Development followed an iterative sprint-based approach incorporating therapist feedback, usability testing, and emphasis on data security and interoperability within the shamiriOS architecture.

Adaptations include (1) migration of legacy patient records from Rafi and SDH to EasyClinic to establish unified clinical histories; (2) integration of real-time therapist availability and appointment scheduling into Rafi’s booking interface, synchronized with the EasyClinic backend; and (3) subscription and plan management enabling users to initiate and manage therapy plans through Rafi with real-time synchronization to EasyClinic. Full technical specifications are in Multimedia Appendix 4.

### Technical Access and Compatibility

All shamiriOS components are web-based or app-based and require no operating system modification, installation privileges, or proprietary software. This was a deliberate design choice to maximize accessibility for nontechnical users and reduce dependency on institutional IT infrastructure across diverse Kenyan implementation settings.

SDH and SPP are web apps accessible via any modern browser on desktop, laptop, or mobile devices. Both incorporate offline-first data capture with automatic synchronization on reconnection. Rafi is a native mobile app available on Android and iOS; core self-help content and check-in features are accessible offline, while therapy booking, triage escalation, and data synchronization require an active internet connection. A supplementary web portal provides browser-based access for users without smartphone access, synced with app data. Table 3 summarizes the minimum technical requirements and offline capabilities for each component.

**Table 3:** Technical access requirements for shamiriOS components^a^.

Component	Access method	Supported browsers or platforms	Device or OS^b^ compatibility	Offline capability
Shamiri Digital Hub	Web app	Chrome 90+, Firefox 88+, Safari 14+, Edge 90+	Desktop, laptop, or mobile—Windows, macOS, Linux, Android, iOS	Partial: offline data capture with automatic sync on reconnection
Rafi	Native mobile app+supplementary web portal	Google Play (Android), App Store (iOS), modern browser (web portal)	Android 8.0+, iOS 12+, web portal via modern browser	Limited: self-help content and check-ins accessible offline; therapy booking, triage, and sync require connectivity
Shamiri Provider Platform	Web app (adapted from EasyClinic)	Chrome 90+, Firefox 88+, Safari 14+, Edge 90+	Desktop, laptop, or mobile—Windows, macOS, Linux, Android, iOS	Not available in current version

^a^All data transmission is encrypted via HTTPS. Authentication uses Open Authorization 2.0 protocols across all components. Full technical specifications are in Multimedia Appendices 2-4.

^b^OS: operating system.

### Engagement Measurement Framework

Engagement with digital health platforms is multifaceted and context-dependent. We adapted a framework defining engagement as the extent to which users interact with each shamiriOS component in ways intended by the design and required for operational or clinical benefit [50]. We distinguish four levels:

1. Initial engagement: account creation, onboarding completion, and first active use.
2. Ongoing engagement: frequency, duration, and consistency of continued use over time.
3. Deep engagement: use of advanced or therapeutically substantive features requiring sustained investment (eg, journaling, clinical triage documentation, and supervisor analytics review).
4. Clinical or operational engagement: actions directly tied to service delivery outcomes (eg, therapy session booking, weekly session attendance logging, and triage escalation).

Engagement is reported separately for each component, reflecting their distinct purposes and user populations:

- SDH (operational or staff-facing): Measured through platform login frequency, data entry compliance rates, feature adoption across user roles, and staff-reported usability and acceptability (single-item satisfaction rating and Net Promoter Score [NPS]). Weekly engagement aligned with supervision and reporting cycles constitutes the target pattern for SDH users.
- Rafi (youth-facing): Measured through registration and onboarding completion (initial engagement), daily and monthly active user (MAU) rates (ongoing engagement), self-guided feature use such as journaling and mood check-ins (deep engagement), and therapy booking and triage escalation (clinical engagement). Episodic or needs-driven engagement may be more clinically appropriate than sustained daily use for this population; we report both frequency-based and action-based metrics.

What constitutes meaningful engagement differs by user type and platform purpose. For youths using Rafi, therapy booking and triage pathway use may be more clinically significant than daily login frequency. For supervisors using SDH, weekly engagement aligned with supervision cycles was operationally sufficient. For clinicians using SPP, session-by-session engagement was anticipated. Engagement data were drawn from backend use logs, session analytics via Metabase, and NPS surveys administered after deployment [51].

### Implementation Evaluation

Our evaluation used pragmatic metrics derived from implementation science instruments [50-53]: measures used were (1) usability—a single-item satisfaction rating on a 1-10 scale (“How easy is the platform to use?”), administered to supervisors and hub coordinators after deployment; (2) acceptability—a NPS item on a 1-10 scale (“How likely are you to recommend this platform to a colleague?”) [51]; (3) engagement—backend use analytics tracking login frequency, feature adoption rates, data entry completeness, and retention over time, reported separately by component; and (4) qualitative feedback—open-ended survey questions and semistructured interviews conducted during and after training sessions.

The choice of pragmatic over standardized measures reflected several constraints. Sprint-based development cycles required rapid, actionable feedback that standard implementation science instruments—which typically require extensive administration time and psychometric analysis—could not reliably provide. The simultaneous development and evaluation of the platform also blurred the boundary between formative user research and summative implementation evaluation, making it difficult to administer standardized instruments at appropriate intervals. Field teams requested brief feedback mechanisms to reduce survey fatigue during intensive deployment.

### Deployment of the shamiriOS Platform

#### Overview

Deployment of SDH and Rafi occurred in phased waves between 2023 and 2024, expanding the user base, geographic coverage, and implementation complexity across waves. SPP deployment is ongoing and is not reported here; a follow-on publication will report SPP deployment and clinical workflow integration separately. Table 4 provides a deployment overview.

**Table 4:** Development of shamiriOS components (Rafi and Shamiri Digital Hub).

Deployment and wave		Details	Time frame	Sites	Size
**Shamiri Digital Hub**					
	Wave 1	Initial deployment in centralized school-based hubs	Q4 2023	5 school-based hubs in Nairobi and Kiambu	358 fellows
	Wave 2	Expansion to decentralized partner sites and diverse implementation contexts	Early 2024	6 additional sites	1312 implementation team members
**Rafi**					
	Rafi 1.0	Limited university pilot with peer counseling groups	2022	7 universities in Kenya	N/A^a^
	Rafi 2.0	Soft-launched across select student clubs	2023	Other universities and colleges	N/A
	Rafi 3.0	Formal deployment at scale through partnership with Mount Kenya University	Early 2024	Mount Kenya University	5000 students

^a^N/A: not applicable.

#### Deployment of the SDH

##### Wave 1: Centralized School-Based Hubs (Q4 2023)

The first SDH deployment targeted 5 school-based hubs in Nairobi and Kiambu counties, selected for high program activity, digital readiness, and established supervisory infrastructure. The goal was to transition day-to-day operations—previously managed through spreadsheets, WhatsApp, and manual logs—to the SDH platform. Training was delivered to 40 supervisors, 6 hub coordinators, and approximately 358 fellows through remote onboarding modules, in-person workshops, and helpdesk support. Each user received a role-based account scoped to their operational responsibilities. Adoption was monitored through backend use metrics and periodic supervisor check-ins, with real-time feedback informing feature refinements.

##### Wave 2: Decentralized Partner Sites (Early 2024)

In early 2024, deployment expanded to 6 additional sites including periurban schools, community centers, and regional nongovernmental organizations operating as local implementation partners. The platform was configured to support site-level customization including localized workflows. Asynchronous training modules, role-specific onboarding videos, and quick-start guides were developed for the more dispersed user base. Site-level implementation champions facilitated peer-to-peer learning beyond formal training periods. Weekly virtual support check-ins were offered during each new site’s first month of onboarding.

#### Deployment of Rafi

##### Rafi 1.0 (2022)

Rafi 1.0 was piloted through partnerships with peer counseling groups at 7 Kenyan universities: the University of Nairobi, Kenyatta University, Jomo Kenyatta University of Agriculture and Technology, Technical University of Kenya, Cooperative University, MKU, and Maasai Mara University. Students were recruited through peer counseling clubs and campus outreach events, then onboarded and asked to provide structured feedback on usability, content tone, and visual design.

##### Rafi 2.0 (2023)

Rafi 2.0 was soft-launched across select student clubs at additional universities and colleges using QR code–based registration and peer ambassador–driven outreach. Students received promotional content and brief onboarding messages introducing Rafi’s core features: wellness check-ins, journaling, triage alerts, and therapy booking.

##### Rafi 3.0—MKU Deployment (Early 2024)

Rafi 3.0 was deployed at scale through a formal partnership with MKU, serving a student population of approximately 25,000, with a targeted deployment cohort of 5000 enrolled students. Deployment included structured digital training for therapists and clinical supervisors covering the provider-facing dashboard and triage flagging protocols; a launch campaign conducted with the university’s student services office and student associations, including peer-led classroom outreach, mental health fairs, and “Rafi Club” engagement events; and a dedicated peer ambassador network tasked with driving registrations. In-app analytics tracked early use trends and informed weekly engagement strategies. Backend dashboards allowed provider teams real-time oversight of flagged users and support queue management.

### Ethical Considerations

The activities described in this paper comprised platform development, an environmental scan of existing digital tools, operational evaluation of the SDH using routine program monitoring data and satisfaction surveys from adult implementation staff, participatory design consultations with university students during Rafi’s development, and aggregated platform use analytics. Per the National Guidelines for Ethical Conduct of Biomedical Research Involving Human Participants in Kenya [54], mandatory ethics review applies to research involving human participants who are “exposed to manipulation, intervention, observation, or other interaction with investigators” or who “become individually identifiable through investigators’ collection, preparation or use of biological material, or medical or other records.” The activities reported here did not meet these criteria: data collection was operational or design-oriented, no sensitive personal or health data were collected, and the intent was product development rather than systematic generation of new knowledge about individuals. Formal ethics committee review was therefore not sought. All data handling complied with Kenya’s Data Protection Act [55] and Shamiri’s institutional data governance framework.

## Results

### Findings From the Environmental Scan

#### Backend Operations Platforms

None of the 6 platforms reviewed met the combined requirements for cost, offline capability, and customizability for task-shifted delivery in Kenya. Salesforce and comparable enterprise systems offered extensive functionality but at licensing costs prohibitive for grassroots organizations. Dimagi CommCare and KoboToolbox offered greater affordability and open-source availability but lacked role-differentiated access, stipend management workflows, and real-time multisite coordination. No platform met all 6 evaluation criteria simultaneously; the highest-scoring alternatives met a maximum of 4 of 6. Full results are in Table 5.

**Table 5:** Review of existing case management and health systems.

Platform	Cost	Ease of use	Customizability	Open source	Ideal use case
Salesforce Health Cloud	High	Moderate	High	No	Care coordination, shared records, team collaboration
Dimagi CommCare	Medium	High	Moderate	No	Mobile-first field data collection
OpenMRS^a^	Low	Moderate	High	Yes	Customizable EMRs^b^ for low-resource clinical settings
Odoo	Medium	High	High	Yes	Operations and resource management
Ona OpenSRP	Low	Moderate	High	Yes	Last-mile community health, offline syncing
Microsoft Dynamics	High	Moderate	High	No	Enterprise health care and operations

^a^OpenMRS: Open Medical Record System.

^b^EMR: electronic medical record.

#### Youth-Facing Digital Mental Health Apps

Of the 28 apps reviewed, 12 were explicitly evidence-based (citing cognitive behavioral therapy, interpersonal therapy, or other clinical frameworks), and 16 were general wellness apps. No app met all evaluation criteria. Common limitations included subscription-based pricing inaccessible to Kenyan university students, the absence of validated clinical content in local languages, no triage or escalation pathway to human providers, and limited or no offline functionality. Only 3 of the 28 apps offered provider-matching features, and none were designed as part of an integrated stepped-care model with backend operational management. Full results are in Table 6, disaggregated by evidence-base status. Further details are in Multimedia Appendix 1.

**Table 6:** Review of existing mental health apps.

Category or feature			Apps with feature (n=28), n (%)		Evidence-based apps only (n=12), n (%)		General wellness apps only (n=16), n (%)
**Evidence-base classification**			—^a^		12 (43)		16 (57)
	Freemium cost model	23 (82.1)		9 (75)		14 (87.5)	
	Subscription only	5 (17.9)		3 (25)		2 (12.5)	
**Provider access**							
	Therapist access	8 (28.6)		7 (58.3)		1 (6.3)	
	Peer support forums	11 (39.3)		4 (33.3)		7 (43.8)	
	AI^b^ chatbot	3 (10.7)		1 (8.3)		2 (12.5)	
**Triage or clinical features**							
	Symptom checker or triage	7 (25)		6 (50)		1 (6.3)	
	Provider matching	5 (17.9)		4 (33.3)		1 (6.3)	
**Self-help resources**							
	Journaling	9 (32.1)		5 (41.7)		4 (25)	
	Meditation	13 (46.4)		6 (50)		7 (43.8)	
	Guided CBT^c^ content	10 (35.7)		10 (83.3)		0 (0)	
**Operational or offline features**							
	Offline functionality	4 (14.3)		1 (8.3)		3 (18.8)	
	Lay-provider or tiered supervision workflows	0 (0)		0 (0)		0 (0)	
	Integrated escalation to in-person care	0 (0)		0 (0)		0 (0)	

^a^Not available.

^b^AI: artificial intelligence.

^c^CBT: cognitive behavioral therapy.

#### Clinical Provider Platforms

Open-source EMR systems including OpenMRS and DHIS2 offered strong clinical documentation features but required substantial technical capacity for implementation and maintenance beyond the reach of most Shamiri implementation partners. EasyClinic was identified as the strongest candidate: it offered client management, teletherapy support, session documentation, appointment scheduling, and analytics dashboards at an accessible cost, with a modular architecture amenable to customization. These findings supported the adaptation of EasyClinic rather than the de novo development of the SPP.

### Current State of shamiriOS

#### Overview

As of early 2025, shamiriOS comprises three interlinked product suites: (1) the SDH, the operational backbone for program management and real-time monitoring; (2) Rafi, a native mobile app (Android and iOS) through which young people access care and mental health resources; and (3) the SPP, an EMR and clinical workflow tool adapted from EasyClinic. SDH and Rafi are fully deployed across multiple centralized and decentralized sites; the SPP is currently in integration testing. Each suite is modular: features can be configured to meet the specific needs of different implementing partners and contexts.

#### Current State of the SDH

##### Roles and Permissions

SDH operates through a role-based permission system aligned with Shamiri’s delivery hierarchy. Five user roles are defined, each scoped to their operational function (Table 7):

- Program administrators hold platform-wide permissions covering multisite oversight, intervention fidelity reporting, stipend management, and global configuration.
- Hub coordinators manage specific hubs, with permissions to assign fellows and supervisors, validate session attendance records, approve stipend submissions, and escalate triage cases.
- Supervisors verify weekly fellow activities, document supervision sessions, and initiate triage referrals for at-risk youths.
- Fellows (lay providers) log session data, track student attendance, and report implementation activities within their assigned hubs. Permissions are currently limited to data entry and personal activity tracking; future enhancements will expand access to include performance dashboards.
- Clinical leads access escalated clinical cases and review triage documentation across all sites.

**Table 7:** Roles and permissions in Shamiri Digital Hub.

Users	Roles and permissions
Program administrators	Possess platform-wide permissions, enabling oversight of multisite activities, intervention fidelity reporting, stipend management, and global settings configuration.
Hub coordinators	Manage specific hubs, with permissions to assign fellows and supervisors, validate session attendance records, approve stipend submissions, and escalate triage cases.
Supervisors	Responsible for the verification of weekly fellow activities, documentation of supervision sessions, and initiation of triage referrals for at-risk youths.
Fellows (lay providers)	Access the platform directly to log session data, track student attendance, and report key implementation activities. Their permissions are limited to data entry and personal activity tracking within assigned hubs. Future system enhancements may expand their access to include feedback tools and performance dashboards to support self-monitoring and reporting efficiency.
Clinical leads	Access escalated clinical cases and review triage documentation across all implementation sites.

Each user interaction is timestamped and logged, supporting full auditability and quality assurance monitoring across the implementation pipeline.

##### Software Architecture

SDH is built on a scalable, microservice-inspired architecture. The frontend is developed in ReactJS and deployed via Vercel’s content delivery network, optimized for performance across variable connectivity environments on desktop and mobile browsers. The backend uses a Node.js runtime interfacing with a PostgreSQL database hosted on Amazon Web Services Relational Database Service; system interactions are managed through a RESTful API framework using JSON Web Tokens for session authentication and encrypted communications. A Metabase analytics layer provides real-time dashboards on session attendance, intervention frequency, supervision compliance, and flagged clinical concerns. Offline-first functionality enables data capture in low-connectivity settings with automatic synchronization on reconnection.

##### Features

The SDH system provides the following features in support of the Shamiri operational model:

- Staff and school management: Dynamic assignment of fellows to school-based groups, tracking intervention session coverage, and historical site performance monitoring.
- Session attendance and tracking: Supervisors enter weekly attendance, attach school visit summaries, and receive automated reminders to ensure data completeness.
- Performance monitoring: Visual analytics dashboards display fellow engagement metrics, session quality ratings, and supervisor oversight reports.
- Financial management: Integrated workflows for stipend submission, validation, and approval through multitiered administrative review pathways.
- Triage and clinical escalation: Supervisors flag students requiring advanced support, triggering structured triage documentation and clinical review workflows.
- Audit trail and compliance monitoring: Comprehensive backend logs maintain records of user interactions, system edits, and data submissions for quality assurance and troubleshooting.

#### Current State of Rafi

##### Roles and Permissions

Rafi’s role and permission system balances user privacy and personalization with clinical oversight. Three roles are defined: (1) users (youths) create and manage personal accounts, complete check-ins, access self-help content, and manage therapy bookings and sharing preferences—all personally identifiable data are encrypted and access-controlled; (2) therapists and clinical staff access a provider dashboard displaying triaged users and appointment requests and may view anonymized user content when the user grants permission; and (3) administrators manage content deployment, update activity flows, and monitor platform use metrics.

##### Software Architecture

Rafi is delivered as a native mobile app (Android and iOS) with a supplementary web portal for users without smartphone access. The frontend is built with the Remix framework, hosted on Vercel, with a responsive design for mobile browsers. The backend uses a hybrid Node.js and Python architecture supporting modular feature development—including AI-generated affirmations and journaling workflows. Structured data (user check-ins and activity logs) are stored in PostgreSQL; media files (eg, journal artwork) are stored in Digital Ocean object storage. Integrations include Google Calendar API for therapist scheduling, Firebase Cloud Messaging for push notifications, and AI services for mood-based artwork generation and personalized reflection prompts. All backend services use HTTPS encryption and Open Authorization 2.0 protocols.

##### Features

Rafi’s feature set is designed to engage youths in a nonclinical but supportive way, supporting well-being monitoring, self-help access, and connection to care:

- Wellness check-ins: Daily and weekly self-ratings across 5 domains (social connection, motivation, academic stress, and others) generating a personalized wellness score and surfacing tailored content.
- AI mood art (Wellness Gallery): Weekly check-in responses are transformed into AI-generated artwork that users can share on the community feed.
- Therapy booking: Users directly schedule individual or group sessions through integrated booking modules connected to therapist availability in the SPP.
- Triage pathways: Algorithmic triggers escalate users to therapist connection workflows based on risk indicators detected during check-ins.
- Community feed: A moderated, anonymous social space where users can share mood art and connect with peers, monitored by peer counselors and the administrator team.
- Reward hub: Gamification elements including badges, streak tracking, and unlockable achievements to promote continued engagement.
- Web portal: A lightweight browser-accessible version providing basic check-in and booking functionality, synced with app data.

Core features—self-guided content (affirmations, journaling, and goal tracking), daily mood check-ins, and well-being score tracking—remain fully functional without internet connectivity. Features requiring server connectivity—therapy booking, AI-generated mood artwork, community feed posts, and data synchronization—queue locally and sync automatically on reconnection.

#### Current State of the SPP

The SPP was adapted from EasyClinic and is currently in integration testing. Formal deployment results are not reported here, as the SPP reached production readiness after the observation period of this study. Adaptations completed to date include migration of legacy patient records from Rafi and SDH to EasyClinic to establish unified clinical histories; integration of real-time therapist availability into Rafi’s booking interface; and subscription plan management enabling users to manage therapy plans through Rafi with synchronization to EasyClinic. The SPP is actively used in Shamiri’s clinic-based services. A dedicated follow-on publication will report SPP deployment, clinical workflow integration, and cross-component data flows.

### Engagement With shamiriOS Components

#### Engagement With the SDH

##### Wave 1 Outcomes (Q4 2023)

By the end of 2023, SDH supported program management across all 5 target hubs, coordinating 358 fellows serving over 20,697 students. Platform acceptability was assessed via postdeployment NPS survey administered to supervisors and hub coordinators (n=22). Respondents reported a mean likelihood of recommending SDH of 8.63 (SD 1.46) and a mean user experience satisfaction score of 8.36 (SD 1.49) on 10-point scales. Qualitative feedback emphasized ease of navigation, the value of real-time data capture, and substantial reductions in administrative workload relative to prior spreadsheet and WhatsApp-based systems. Stipend workflow automation and centralized fellow attendance tracking were the most frequently cited valued features (Multimedia Appendix 3).

##### Wave 2 Outcomes (Q1 2024)

By the end of Q1 2024, SDH supported 1195 fellows, 111 supervisors, and over 76,344 students across all active hubs—representing a 3.3-fold increase in fellows, a 2.8-fold increase in supervisors, and a 3.7-fold increase in student reach relative to wave 1. Ongoing monitoring included automated alerts for low engagement, biweekly platform review meetings with operations staff, and structured feedback channels informing subsequent sprint planning cycles (Multimedia Appendix 3).

#### Engagement With Rafi

##### Overview

Rafi engagement is reported across 4 dimensions: initial engagement (registration and onboarding), ongoing engagement (MAU rates), deep engagement (self-guided feature use), and clinical or operational engagement (therapy booking and triage escalation). As noted in the Methods section, episodic or needs-driven engagement may be more clinically meaningful than sustained daily use for a youth mental health platform; we report both frequency-based and action-based metrics.

##### Rafi 1.0—Initial Pilot (2022)

Of the 1349 students who registered interest, 857 (63.5%) enrolled as active users by the end of 2022. Among enrolled users, 74% (634/857) engaged with the self-guided digital wellness courses—the platform’s highest-engagement feature. In total, 37.1% (318/857) booked at least 1 therapy session, and 33% (105/318) of those who booked attended.

##### Rafi 2.0—Expanded Deployment (2023)

During the 2.0 deployment, 1008 students registered accounts. Therapy booking improved markedly: 50.4% (508/1008) of users booked at least 1 session (compared to 318/857, 37.1% in version 1.0), and 44.9% (228/508) of those attended (compared to 105/318, 33%). Self-guided wellness module engagement remained high at 77.3% (779/1008). Monthly active engagement declined significantly after the first 4 weeks, however—a persistent attrition pattern that directly informed the gamified re-engagement architecture of Rafi 3.0.

#### Rafi 3.0—Institutional Deployment, MKU (2024)

##### Initial Engagement

Between January and September 2024, a total of 3737 of the 5000 targeted MKU students registered on Rafi—a 74.7% initial registration rate and the largest single-cohort registration in the platform’s deployment history.

##### Ongoing Engagement and Attrition

Despite strong initial registration, MAU rates declined significantly across the 9-month observation window—from 7.4% in January 2024 to 0% by September—indicating near-complete disengagement among the registered cohort by the end of the observation period. Daily active user rates showed a consistent downward trend from the weeks following launch.

##### Feature-Specific Engagement

Across the observation period, 3.8% (n=142) of registered users used the daily check-in tool, 1.5% (n=56) used journaling features, 0.5% (n=20) booked therapy sessions, and 0.4% (n=15) used the Ask a Therapist chat feature. Affirmations and other low-touch tools demonstrated limited traction (<0.2%). An NPS survey administered in June 2024 (n=275) found that 15% (n=41) of respondents had attended at least 1 therapy session; among users who engaged with self-guided tools, 99.4% (n=141) subsequently booked therapy appointments.

###### Implementation Barriers and Facilitators: Preliminary Observations

The following observations were documented through operational feedback channels—training session notes, helpdesk tickets, monthly check-in meetings, NPS survey comments, and sprint planning discussions (Multimedia Appendices 2 and 3).

#### Barriers

##### Internet Connectivity

Inconsistent connectivity in periurban schools and community centers caused data synchronization failures and incomplete session logging in SDH. Supervisors at some sites reported traveling to areas with stronger connectivity to submit weekly reports, reducing data timeliness. For Rafi, the connectivity requirement for therapy booking and triage constrained clinical pathway use among students at lower-resourced sites.

##### Device Constraints

Not all fellows had personal smartphones capable of running modern browser-based apps; device sharing at some hubs created bottlenecks for timely data entry. For Rafi, storage constraints on student smartphones contributed to postregistration uninstalls—limited phone storage was cited in helpdesk and peer ambassador feedback as a contributing factor.

##### Engagement Infrastructure Readiness

The MKU deployment of Rafi 3.0 launched before its engagement infrastructure was fully operational. Push notification workflows, in-app communication features, and the gamification reward system were not functioning as intended at launch. This prevented timely re-engagement of users experiencing early drop-off and left the team without the real-time use tracking needed to respond to emerging attrition patterns.

##### Ambassador Incentivization Misalignment

The peer ambassador model at MKU incentivized students financially based on registration counts, producing high initial sign-ups but not the values-based peer promotion needed to drive sustained engagement. Postlaunch operational review found that peer counselors—who combined mental health training with authentic relational credibility—were substantially more effective at converting registrations into active users than financially incentivized ambassadors.

##### Absence of Institutional Integration

At MKU, the absence of a formal Memorandum of Understanding with university leadership limited institutional buy-in and delayed clinical pathway activation. Caregivers and clinical staff were not fully onboarded at the time of the student launch, meaning therapy booking and triage escalation features were not immediately functional during the highest-engagement window following sign-up.

#### Facilitators

##### Alignment With Existing Workflows

SDH adoption was strongest at sites, where the platform mirrored and enhanced existing workflows rather than replacing them entirely. Users at these sites reported reduced learning curves and described the platform as intuitive to adopt.

Role-Based Access Design

The role-differentiated account structure in SDH was consistently cited as a facilitator. Users received dashboards and input fields scoped to their specific responsibilities, reducing cognitive overload and increasing the perceived relevance of the platform to day-to-day tasks.

##### Peer Counselor-Led Outreach

Peer counselors with mental health training and established peer relationships demonstrated significantly higher engagement conversion than incentivized ambassadors. This has directly informed the revised outreach strategy for subsequent Rafi deployments, shifting from large launch-event acquisition toward gradual rollout through trusted peer networks.

##### Free Access Model

The absence of licensing fees for both SDH and Rafi was consistently identified by implementation partners as a critical enabler, removing the cost barrier that had prevented uptake of commercially licensed platforms reviewed in the environmental scan.

##### Offline-First Functionality in SDH

The ability to capture session data without active internet connectivity was consistently highlighted by supervisors at periurban sites as a key facilitator, reducing dependency on stable connectivity for core operational data entry.

## Discussion

### Principal Findings

This study documents the development and early deployment of shamiriOS, an open-source, modular digital platform for scalable, task-shifted youth mental health service delivery in Kenya [15,20,23]. Three principal findings emerge.

First, an environmental scan confirmed that no existing platform adequately met the combined operational, clinical, and youth engagement requirements of the Shamiri stepped-care model. Reviewed platforms failed on at least 2 of 6 evaluation criteria—most commonly cost, offline capability, and customizability for task-shifted workflows. This reflects not a critique of those platforms, but confirmation that the infrastructure gap in community-based mental health delivery in Kenya was real and unaddressed by the global product landscape [34].

Second, the SDH demonstrated strong early feasibility and acceptability. Deployed across 5 hubs by the end of 2023 and expanding to 11 sites supporting over 76,344 students by Q1 2024, SDH replaced fragmented operational systems with real-time, role-differentiated digital infrastructure. Implementation staff reported high satisfaction (usability: mean 8.36, SD 1.49; NPS: mean 8.63, SD 1.46), and qualitative feedback consistently identified reductions in administrative burden and improvements in data timeliness and completeness. These results are consistent with evidence that well-designed operational platforms improve efficiency and oversight capacity in task-shifted delivery systems in LMICs [29,31,35-37,56].

Third, Rafi demonstrated strong initial reach—74.7% (n=3737) registration among the targeted MKU cohort—but significant engagement attrition, with MAU rates declining from 7.4% in January 2024 to near 0 by September. This pattern is common in digital mental health globally [37,39,57-60], but operational analysis identified specific, modifiable causes beyond structural or population-level explanations [39,58-60]. Among users who engaged with self-guided features, 99.4% subsequently booked therapy appointments—a cross-sectional finding requiring cautious interpretation, but one suggesting that self-help engagement may function as a gateway to clinical help-seeking for those who remain on the platform.

### shamiriOS in the Context of Existing Digital Mental Health Platforms

shamiriOS contributes to a growing landscape of digital mental health platforms in LMICs [29-31,35-37]. What distinguishes it is not any single feature, similar apps exist, but 3 architectural characteristics that together address gaps in the current platform landscape [61,62].

First, shamiriOS integrates operational management (SDH), youth engagement (Rafi), and clinical workflows (SPP) within a single interoperable ecosystem. Most existing platforms address one of these functions; few attempt integration across all 3. This reflects our view that sustainable digital mental health infrastructure must support not just the end-user experience, but the full organizational and clinical system within which that experience is embedded [63,64].

Second, the platform uses a modular, component-based design in which each suite operates as an independent service with its own codebase, database, and deployment pipeline, connected through defined API contracts. An implementing organization could adopt SDH without deploying Rafi or adapt Rafi for a different clinical context without requiring the full SDH infrastructure. RBAC is configurable per deployment—the same instance can serve a secondary school program with 3 user roles or a university-based stepped-care service with 10—without code changes. This modularity is deliberate: no 2 implementation contexts share identical operational requirements, and platform rigidity is a common reason digital health tools fail when transferred across settings [63,64].

Third, the open-source architecture—built on Node.js, PostgreSQL, and React, with RESTful APIs and a phased Fast Healthcare Interoperability Resources compliance roadmap—lowers the barrier to adaptation by other technical teams [63-65]. Offline-first data capture, customizable reporting, and flexible intervention workflows are configurable features rather than hardcoded assumptions [63-65].

To the best of our knowledge, no comparable integrated open-source platform has been developed and deployed for community-based stepped-care mental health delivery in Kenya or comparable East African settings. We make no claim to novelty for the global landscape, where numerous digital mental health platforms exist [30,31,35]. The contribution is contextual: a platform built from the ground up for the operational, cultural, and infrastructural realities of implementation in Kenya and similar low-resource settings.

Extending shamiriOS to nonmental-health domains is a plausible future direction, but currently hypothetical. The modular architecture could, in principle, support other task-shifted, community-based delivery programs, but such extensions would require domain-specific user research and evaluation rather than direct platform transfer.

We believe publishing shamiriOS as open source serves an important purpose: making the architecture and code available for other teams to adapt, critique, and build upon. The precedent set by OpenMRS and DHIS2—adapted across dozens of countries while maintaining core architectural integrity—suggests that platform-level adaptability is achievable when accompanied by community-led localization [65-68]. Implementation toolkits, training modules, and documentation are being developed to lower the barrier for organizations in Kenya and comparable settings, with the explicit expectation that substantial localization will be required.

### shamiriOS Can Support Backend Operations Within Task-Shifted Youth Mental Health Systems

The SDH was feasible and acceptable across both centralized and decentralized implementation contexts. The transition from fragmented workflows—spreadsheets, manual stipend logs, and WhatsApp-based supervision coordination—to a unified real-time platform was achieved across 5 hubs in Q4 2023, expanding to 11 sites supporting 1195 fellows, 111 supervisors, and over 76,344 students by Q1 2024. A 3.7-fold increase in student reach within a single year, maintained alongside high satisfaction scores, suggests that the platform can scale without deterioration in usability or staff experience. An NPS of 8.63 and a usability rating of 8.36 are consistent with evidence that well-designed operational platforms improve efficiency and oversight capacity in task-shifted delivery systems [30,31,35,56]. Qualitative feedback highlighted 3 valued features: centralized real-time visibility of fellow attendance across sites; automated stipend workflows, which reduced manual verification errors; and Metabase analytics dashboards, which allowed nontechnical supervisors to interrogate program data without developer support. The last is particularly relevant for scale: building analytical self-sufficiency into the platform reduces long-term dependency on the central technical team [30,31,35,56].

Operational feedback from the wave 2 expansion revealed several implementation-relevant patterns. Adoption was strongest at sites where SDH mirrored and enhanced existing workflows rather than replacing them wholesale—consistent with implementation science evidence that technology adoption is smoother when tools augment rather than disrupt established practice [31,64]. Device sharing at some hubs created bottlenecks in timely data entry, temporarily reducing completeness in the first weeks of deployment. Offline-first data capture—introduced in version 3—addressed connectivity constraints but not device access. Future deployments to settings with low device-to-user ratios may need to treat hardware access as a precondition for adoption or consider lighter-weight interfaces optimized for brief interactions on shared devices.

### Engagement Is a Challenge for Youth-Facing Apps Like Rafi

The gap between registration and sustained engagement was striking. High initial registration confirms that broad uptake is achievable through institutional partnerships and peer-led outreach. The subsequent decline—with sustained active use falling well below the 4% at 6 months common in reviews of consumer mental health apps [39,58]—is consistent with the literature but requires careful disaggregation.

Qualitative feedback identified several contributing factors: low perceived ongoing need among students who registered out of curiosity during launch, feature overload from multiple simultaneous engagement pathways, device storage constraints, and notification fatigue. These represent different problems requiring different solutions—notification calibration addresses fatigue but does nothing for users with genuinely low perceived need or stigma concerns [69-71].

More structurally, Rafi was designed around continuous self-monitoring—daily check-ins, journaling, and affirmations—which requires users to perceive ongoing value in routine self-assessment. For a predominantly healthy student population, this model may not fit typical help-seeking patterns, which tend to be episodic and crisis-driven rather than habitual [69-71]. Future iterations should consider whether a shift toward episodic engagement—designed for crisis-prompted access to care rather than daily wellness tracking—would better match actual user needs [39].

The finding that 99.4% of Rafi users who engaged with self-guided tools subsequently booked a therapy appointment—though cross-sectional and not causal—suggests that for students who do engage, the platform functions effectively as a gateway to higher-intensity care [69,71]. This points to a segmented engagement strategy: prioritizing acquisition and early retention for students with active mental health needs, rather than sustained mass engagement across the full student population.

### shamiriOS Features in Development

#### Overview

Ongoing development is guided by field feedback, platform analytics, and emerging implementation needs [18,20,23,72,73].

SDH is being upgraded for more robust multisite operations, with planned enhancements including automated triage escalation for flagged students, expanded school and hub-level reporting, credential management, and intervention quality dashboards [18,23]. Standardized onboarding and role-based interfaces are being embedded to support consistency, as the platform scales to new partner organizations [18,23].

The next version of Rafi is designed to address the engagement challenges described earlier. Design priorities include gamified onboarding sequences, mood-responsive motivational content, and more direct pathways connecting students to therapists. Personalized content journeys adapted to user behavior are intended to reduce early drop-off [32,74,75]. The interface is being restructured to reduce feature overload, beginning with a simpler onboarding pathway before exposing users to the full feature set. Rafi’s architecture is also being refactored for tighter SPP integration, supporting seamless transitions from self-guided to provider-supported care [30,32].

SPP is not reported as a deployed component in this study by design: it reached production readiness after the observation period, and its deployment and clinical integration merit are ongoing. Still, SPP integration continues with development priorities including real-time therapist scheduling, digital session documentation, and bidirectional data flow between clinical and operational layers. Historical client data migration is in progress to ensure continuity of care across platforms [33,34].

#### Future Development: AI Integration

A key area of planned development is the integration of AI tools—referred to internally as shamiriAI—across 3 priority domains: AI-assisted provider training and supervision, including automated fidelity scoring of intervention sessions; precision-matching of users to content and care levels based on symptom severity and engagement behavior; and symptom network analysis to inform modular intervention design. The potential benefits—particularly for supervision at scale, where human oversight capacity is a binding constraint in task-shifted delivery—are significant [31,76].

AI integration in mental health carries well-documented risks that are amplified in the Kenyan context [77-80]. These include dangerous clinical advice from language models, inappropriate substitution of AI for human therapeutic relationships, exploitation of sensitive personal data, and perpetuation of biases embedded in training datasets [77,81,82]. Dominant large language models are trained overwhelmingly on English-language [83], Western psychological literature [84,85], creating fundamental misalignment with Kenyan contexts where the experience of mental health may differ from Western models [86-90]. Regulatory frameworks in most African countries have not kept pace with the speed of AI deployment [77,78,91].

For these reasons, shamiriOS approaches AI integration as a learning-first rather than deployment-first activity. Guiding principles include an “AI-in-the-loop” design retaining human agency over care; training data grounded in African languages and contexts; community oversight involving youths, fellows, and clinicians as reviewers rather than only end users; strict data governance; and prospective safety monitoring. shamiriAI is presented as a set of research questions to be answered through rigorous evaluation. Future phases will assess feasibility, acceptability, cultural appropriateness, and safety before any AI feature enters service delivery [77].

### Data Protection and Engagement Considerations

shamiriOS was designed to improve access to youth mental health services, but digital data collection introduces risks requiring explicit acknowledgment. Kenya’s Data Protection Act (2019) provides a legal framework [91], but enforcement capacity is limited, and the gap between legal protection and practical data security in community-based implementations remains significant [92-94].

Current data protection measures include end-to-end HTTPS encryption, data deidentification, RBACs, separation of operational data (SDH) from clinical and personal data (Rafi and SPP), secure cloud hosting with encrypted databases, and Open Authorization 2.0 authentication. Rafi does not collect passive sensor data. Anonymous community feed features allow engagement without linking posts to clinical data.

These measures reduce but do not eliminate risk [92-94]. Future work will strengthen protections through user-controlled data deletion and full data export; transparent disclosure about what data are collected, how they are stored, and scenarios under which they could be accessed by third parties; and, where technically feasible, local device storage options that reduce cloud dependency [92-94]. shamiriOS’s data governance policies will be published openly as part of the open-source repository to enable external scrutiny.

shamiriOS was developed through a user-centered design process [40,41]—not community-led development. End users informed design decisions as consultants and testers; strategic and architectural decisions were made by Shamiri’s technical and clinical leadership. This reflects pragmatic constraints of real-world development and believe that the level of inclusion on feature development, content priorities, and data governance is appropriate for platforms at this stage. Future governance structures should consider greater community ownership [42,45].

### Limitations

Several limitations should inform the interpretation of these findings. The environmental scan was structured but not exhaustive. Proprietary, region-specific, or non-English–language platforms may not have been captured, and the scan assessed market availability and feature coverage rather than clinical efficacy; it should not be read as a systematic review.

We did not use validated implementation science measures for feasibility, acceptability, or usability. Pragmatic industry metrics—NPS, MAU rates, and feature adoption rates—provided actionable feedback for development sprints but lack the reliability and cross-study comparability of instruments such as the System Usability Scale [95], Acceptability of Intervention Measure [53], or Feasibility of Intervention Measure [53]. Future evaluations of the stable platform should use these standardized instruments.

Barriers and facilitators were documented through operational feedback channels—training sessions, helpdesk tickets, monthly check-ins, and NPS qualitative responses—rather than formal implementation research using validated frameworks such as Consolidated Framework for Implementation Research or Exploration, Preparation, Implementation, Sustainment [96,97]. Our understanding of why engagement varied across sites and user groups is therefore incomplete. Future studies must prioritize rigorous implementation science methods.

Deployment results—particularly Rafi’s MKU engagement data—reflect a 9-month observation window. Longitudinal engagement patterns, clinical outcomes, and cost-effectiveness at scale have not been assessed. The 99.4% therapy-booking rate is a cross-sectional observation that cannot establish causality.

Finally, shamiriOS used user-centered design with consultation, not fully participatory or community-led design. Future studies should consider a full participatory approach and investigate the implications of different approaches on cultural grounding, local ownership, and long-term sustainability.

### Conclusions

The development and early deployment of shamiriOS demonstrate the feasibility of modular, open-source digital infrastructure for scalable, task-shifted youth mental health service delivery in Kenya. SDH showed strong feasibility and acceptability at scale, with satisfaction scores comparing favorably with digital health benchmarks and a 3.7-fold expansion in reach over a single year. Rafi demonstrated broad initial reach but surfaced important, actionable lessons about the conditions required for sustained engagement—lessons specific enough to have directly informed deployment strategy for future cohorts.

shamiriOS is not a finished product. Its full value will only be realized once all 3 components are deployed, integrated, and rigorously evaluated. The evidence here establishes a foundation: the core infrastructure works, it is valued by its users, and the most significant obstacles to impact lie not in platform design but in implementation readiness, incentive structures, and institutional integration—all of which are addressable.

We envision shamiriOS as an open-source, contextually grounded infrastructure for decentralized mental health service delivery in Kenya, available for adaptation by teams in comparable settings—with adaptation requiring necessary contextual redesign. By publishing both the platform and an account of its early deployment, we aim to contribute not only a technical resource but a set of implementation lessons to the broader community working to close the youth mental health treatment gap in Kenya and beyond.

## Appendix

Multimedia Appendix 1 Environmental scan: methodology and reviewed platforms.

Multimedia Appendix 2 Rafi: co-design methodology and version history.

Multimedia Appendix 3 Shamiri Digital Hub: technical architecture, development, and deployment outcomes.

Multimedia Appendix 4 Shamiri Provider Platform: EasyClinic adaptation and Fast Healthcare Interoperability Resources compliance roadmap.

## Abbreviations

AI: artificial intelligence
API: application programming interface
DHIS2: District Health Information System 2
EMR: electronic medical record
LMIC: low- and middle-income country
MAU: monthly active user
MKU: Mount Kenya University
NPS: Net Promoter Score
OpenMRS: Open Medical Record System
RBAC: role-based access control
REST: Representational State Transfer
SDH: Shamiri Digital Hub
SPP: Shamiri Provider Platform
