# Supplementary material for: Early Deployment of an Integrated Digital Platform (shamiriOS) for Scalable Youth Mental Health Service Delivery in Kenya: Development and Usability Study
Source: JMIR Hum Factors. 2026 Jun 3;13:e79107. doi: 10.2196/79107 (PMC13276469; doi:10.2196/79107)
Supplement: Multimedia Appendix 1 [file humanfactors_v13i1e79107_app1.docx]

More information, including access to underlying code, can be found on this Open Science Framework repository.

For more information, please contact Shadrack Lilan ([shadrack.lilan@shamiri.institute](mailto:shadrack.lilan@shamiri.institute))

# Environmental Scan: Methodology and Reviewed Platforms

## Overview

Between May and July 2023, we conducted a two-part environmental scan to assess the suitability of existing digital tools for supporting Shamiri’s stepped-care model. The scan covered: (1) backend case management and health systems used in global health and social service delivery contexts; and (2) youth-facing digital mental health applications available on consumer app stores.

This environmental scan was a structured market assessment of digital platforms relevant to stepped-care, community-based youth mental health delivery in Kenya. It assessed market availability, feature coverage, and contextual fit. [NEW] It was not a systematic review of clinical efficacy and should not be read as such. Rather, it represented a pragmatic assessment of whether existing solutions could meet the operational, clinical, and user-engagement requirements of the shamiriOS architecture without substantial customization.

All platforms were assessed against the criteria in Table S1 below.

| **Criterion** | **Justification** |
| --- | --- |
| Cost | Must be affordable or free for an NGO-led implementation at scale in Kenya |
| Ease of Use | Must be usable by lay providers, supervisors, and adolescent clients with varying levels of digital literacy |
| Customizability | Must accommodate task-shifting workflows, school-based implementation, and stepped-care triage |
| Open-Source Availability | Ensures accessibility, adaptability, and long-term sustainability without vendor lock-in |
| Suitability for Mental Health | Requires support for psychosocial content, triage workflows, and group or individual care formats |
| Low-Resource Appropriateness | Must support offline use, low-bandwidth environments, and options for local hosting |

## Review 1: Case Management and Health Systems

### Scope and Approach

Between May and July 2023, we conducted a desktop review of six global case management and health information platforms through a combination of public documentation, product demonstration sessions, and technical repository review. Platforms were identified through searches of published global health implementation literature, digital health platform directories (e.g., Digital Square’s Global Goods Guidebook), and recommendations from the broader global mental health technology community.

### Platforms Reviewed

- *Salesforce Health Cloud* — a commercial CRM-based health management platform
- *Microsoft Dynamics 365* — a commercial enterprise workflow platform with health modules
- *OpenMRS* — an open-source electronic health record system widely deployed in global health settings
- *CommCare* — a mobile-first case management platform designed for community health workers
- *RapidPro* — an open-source platform for managing messaging and workflow automation in health and development programmes
- *DHIS2* — a national-scale health information system used in numerous African countries

### Summary of Findings

**Cost:** Commercial platforms (Salesforce, Microsoft Dynamics 365) were prohibitively expensive for NGO implementation at scale. Open-source platforms (OpenMRS, CommCare, RapidPro, DHIS2) offered free-to-low-cost deployment models.

**Customizability for Mental Health:** Most platforms were designed for general health service delivery. Mental health-specific workflows (stepped-care triage, psychosocial supervision, peer support documentation) required substantial customisation across all platforms reviewed.

**Low-Resource Appropriateness:** CommCare and RapidPro offered mobile-first architectures suitable for low-bandwidth environments. OpenMRS and DHIS2 required more robust server infrastructure. None of the reviewed platforms offered offline-first data capture as a core feature.

**Open-Source Status:** Four of six platforms reviewed were open-source (OpenMRS, CommCare, RapidPro, DHIS2), providing a foundation for customisation. However, each required significant engineering investment to adapt for the specific shamiriOS use case.

**Conclusion:** No single platform adequately met all six evaluation criteria for shamiriOS requirements. The decision was made to build custom components (SDH for operational management and Rafi for youth engagement) rather than adapt existing platforms.

## Review 2: Youth-Facing Digital Mental Health Applications

### Search Strategy

We conducted a targeted search of digital mental health applications available on the Google Play Store and Apple App Store between June and July 2023. Search terms included: “mental health,” “therapy,” “counselling,” “anxiety,” “depression,” “mindfulness,” “youth mental health,” and “adolescent mental health.” We also reviewed published app assessment studies (e.g., from the Research and Development Centres in Digital Mental Health programme at Kings College London) to identify additional high-quality apps for evaluation.

### Eligibility Criteria

Applications were included if they: (1) targeted adolescents or young adults (13–25 years); (2) addressed mental health, wellbeing, or psychological support; (3) included at least one of the following: self-guided interventions, therapist booking/communication, or peer support features; and (4) were available in English and accessible to Kenyan users via standard app stores.

Applications were excluded if they: (1) required direct clinical diagnosis or medication management; (2) were discontinued or no longer actively maintained; (3) were restricted to specific geographic regions excluding Kenya; or (4) focused exclusively on physical health or other non-mental-health domains.

### Assessment Framework

Each application was assessed on four dimensions:

1. **Feature Coverage:** presence of self-guided features, therapist booking, peer support, offline functionality, and assessment/tracking tools
2. **User Experience Design:** visual design, navigation clarity, content tone, and appropriateness for adolescent users
3. **Clinical Integration:** compatibility with stepped-care workflows, therapist-client communication, and outcome measurement
4. **Accessibility for Low-Resource Contexts:** offline functionality, data privacy, storage footprint, and suitability for low-bandwidth environments

### Summary of Findings (28 Apps Reviewed)

Twenty-eight applications were systematically assessed. The following summary reflects key findings:

| **Feature Domain** | **Apps with Capability** | **Percentage** |
| --- | --- | --- |
| Self-guided content (articles, journaling, mood tracking) | 24 | 86% |
| Therapist booking or direct therapist communication | 11 | 39% |
| Peer support or community features | 8 | 29% |
| Offline functionality (any features usable without connectivity) | 5 | 18% |
| Assessment instruments (PHQ-9, GAD-7, or equivalent) | 14 | 50% |
| Explicit stepped-care or tiered intervention design | 3 | 11% |
| Cost: Free or freemium model | 19 | 68% |
| Open-source code availability | 2 | 7% |

### Key Findings:

- **Self-guided features were prevalent** but often designed for high-literacy, high-engagement users with continuous connectivity.
- **Therapist integration was limited:** Most apps with therapist features required users to book through external platforms or clinic referral systems, limiting clinical integration.
- **Offline functionality was rare:** Only 5 of 28 apps (18%) offered any features usable without internet connectivity — a critical gap given the intermittent connectivity common in Kenyan school and university settings.
- **Stepped-care design was absent:** Only 3 apps explicitly incorporated stepped-care logic (initial self-help screening, then triage to appropriate care level). Most offered a linear, one-size-fits-all intervention approach.
- **Peer support was under-represented:** Only 8 apps included peer support features, and most required external platform management rather than integration with clinical care.
- **Open-source availability was extremely limited:** Only 2 of 28 apps were fully open-source, limiting long-term customisation and sustainability for Kenyan implementation.

**Conclusion:** While the reviewed apps represented high-quality, well-designed digital mental health tools, none adequately integrated the combination of self-guided features, therapist communication, peer support, offline functionality, and stepped-care logic required by shamiriOS. This confirmed the rationale for developing custom youth-facing technology rather than adapting an existing commercial or open-source application.
