# Supplementary material for: Early Deployment of an Integrated Digital Platform (shamiriOS) for Scalable Youth Mental Health Service Delivery in Kenya: Development and Usability Study
Source: JMIR Hum Factors. 2026 Jun 3;13:e79107. doi: 10.2196/79107 (PMC13276469; doi:10.2196/79107)
Supplement: Multimedia Appendix 2 [file humanfactors_v13i1e79107_app2.docx]

# Rafi Co-Design Philosophy and Framework

Rafi was developed through a user-centred design process with direct input from young people, peer counsellors, and implementation partners. The design philosophy incorporated five core principles:

1. **Youth-Centredness:** Design decisions prioritised the preferences, language, and cultural context of young Kenyan users over global mental health app conventions.
2. **Stigma-Awareness:** All design choices were evaluated for potential to reinforce mental health stigma. This led to decisions like anonymous community features and non-clinical interface language.
3. **Stepped-Care Integration:** Features were designed to seamlessly route users to appropriate care levels based on symptom severity and preference, not as a linear one-size-fits-all intervention.
4. **Accessibility for Varying Digital Literacy:** The interface was designed for users with a wide range of smartphone experience, including those with limited prior app usage.
5. **Offline Functionality:** Core self-guided features were built to be fully functional without internet connectivity, reflecting the reality of intermittent connectivity in Kenyan schools and universities.

# Co-Design Process by Version

## Rafi 1.0 (May 2022): Initial Prototype

**Scope:** A proof-of-concept mobile app testing core concepts of mood tracking and self-guided content delivery.

**Design Process:** - Initial research: Semi-structured interviews with 12 young people (18–24 years) at Shamiri partner organisations exploring preferences for mental health support features and app design - Iterative design: 4 rounds of low-fidelity prototype testing with 8–10 young people per round, using paper prototypes and Figma wireframes - Key design decisions: Simple, single-screen mood check-in; warm, conversational tone in app copy; option to create anonymous user accounts

**Outcome:** A functional prototype validating the concept that young people in Kenya would engage with a self-guided mental health app and confirming preference for anonymous and non-clinical design.

## Rafi 2.0 (Early 2023): Co-Creation and Stable Platform

**Scope:** Full platform with self-guided features, therapy booking, peer support, and offline functionality. This was the first version deployed at scale.

**Design Process:**

- **Foundational research:** 18 focus groups (n=2–4 participants each) with university students across three Kenyan universities (University of Nairobi, Kenyatta University, Mount Kenya University) exploring mental health help-seeking barriers and preferences for digital support
- **Co-creation sessions:** [NEW] 77 university-aged youth across three institutions participated in structured co-creation workshops. Sessions were organised around four core activities:
  - **Card-sorting exercises:** Participants organised 25 proposed app features into priority categories (must-have, nice-to-have, not needed). Analysis identified that therapy booking and mood tracking were universally rated must-have; peer support and gamification features were polarised
  - **Prototype walkthrough activities:** Participants navigated low-fidelity wireframes of the complete app flow (onboarding, mood check-in, content library, therapy booking). Feedback focused on navigation clarity, content tone, and visual design appropriateness
  - **Pain-point mapping workshops:** Participants identified specific barriers to mental health help-seeking (stigma, cost, accessibility, trust in providers). Designers mapped how app features could directly address each barrier
  - **Feature prioritisation voting:** Participants ranked 15 proposed features using a cumulative voting system. Results informed the final feature set for Rafi 2.0
- **Design decisions emerging from co-creation:**
  - **Anonymous community feed:** Directly addressed stigma concerns identified in pain-point mapping. Users could post struggles and receive peer support without revealing identity
  - **Mood-to-artwork feature:** Identified in card-sorting as a creative, non-clinical engagement hook that would appeal to users reluctant to engage with traditional mental health terminology
  - **Peer-like tone in app copy:** Participants consistently requested “warm, like talking to a friend” language rather than clinical terminology. This drove the decision to write all in-app text in conversational Kenyan English
  - **Simplified onboarding:** Testing revealed that detailed assessment questionnaires at the outset created friction. Final design included minimal onboarding (name, preferred pronouns, consent to terms) before users could access core features
- **Iterative design testing:** 12 rounds of usability testing with 5–8 young people per round, incorporating feedback into each sprint

**Outcome:** A fully functional, extensively tested platform deployed to 275 students at Mount Kenya University’s initial launch event.

## Rafi 3.0 (Early 2024): Institutional Integration and Post-Deployment Iteration

**Scope:** Enhanced version incorporating feedback from the MKU deployment and two earlier university cohorts (School of Public Health cohort n=89; Kenya Red Cross cohort n=145).

**Design Process:**

- **Rafi 3.0 user testing:** [NEW] The MKU deployment provided extensive real-world usage data. Design improvements were informed by multiple feedback channels:
  - NPS survey responses (n=275): Qualitative feedback on usability, features that helped most, barriers to sustained engagement
  - Helpdesk ticket analysis (n=412 total tickets): Systematic review of user-reported bugs, feature requests, and confusion points
  - Peer ambassador debriefs: Monthly meetings with student peer counsellors identifying which features drove engagement and which created confusion
  - Structured feedback sessions with MKU student leadership (n=8 students): In-depth exploration of engagement barriers and feature preferences
- **Key design changes informed by deployment feedback:**
  - **Improved onboarding flow:** Users reported confusion about the therapy booking workflow. V3.0 redesigned the path from mood check-in to therapy booking with explicit prompts and clearer explanation of what booking entailed
  - **Enhanced push notification configurability:** Users wanted to control engagement frequency. V3.0 introduced granular notification settings allowing users to choose daily check-in reminders, feature discovery prompts, or no prompts at all
  - **Optimised app storage footprint:** Device storage constraints were cited in helpdesk analysis and ambassador feedback as a contributing factor to uninstalls. V3.0 reduced the app size from 142 MB to 89 MB through code optimisation and dynamic content loading
  - **Added mood history visualisation:** Users requested the ability to see trends in their mood over time. V3.0 added a simple graph showing mood scores over the past 30 days

**Outcome:** An enhanced platform with improved onboarding, engagement flexibility, and storage efficiency, ready for deployment at additional sites.

# Rafi Version Feature Summary

| **Feature** | **Rafi 1.0** | **Rafi 2.0** | **Rafi 3.0** |
| --- | --- | --- | --- |
| **Self-Guided Features** |  |  |  |
| Mood tracking (daily check-in) | ✓ | ✓ | ✓ |
| Affirmations and positive prompts | ✗ | ✓ | ✓ |
| Journal/reflection prompts | ✗ | ✓ | ✓ |
| Goal tracking and progress updates | ✗ | ✓ | ✓ |
| Psychoeducational content library | ✗ | ✓ | ✓ |
| Mood-to-artwork (AI feature) | ✗ | ✓ | ✓ |
| Offline functionality for above | ✗ | Partial | Full |
| **Clinical Engagement** |  |  |  |
| Therapy booking system | ✗ | ✓ | ✓ |
| Appointment reminders | ✗ | ✓ | ✓ |
| Therapy session summary feedback | ✗ | ✗ | ✓ |
| **Peer Support** |  |  |  |
| Anonymous community feed | ✗ | ✓ | ✓ |
| Peer response system | ✗ | ✓ | ✓ |
| **System Features** |  |  |  |
| Offline mood/journal sync on reconnection | ✓ | ✓ | ✓ |
| Push notifications | ✗ | ✓ (limited) | ✓ (configurable) |
| Analytics dashboard (admin view) | ✗ | ✓ | ✓ |
| Multi-language support | English only | English, Kiswahili | English, Kiswahili |
| Available platforms | Web (Figma prototype) | Android, iOS | Android, iOS |
