# Supplementary material for: Early Deployment of an Integrated Digital Platform (shamiriOS) for Scalable Youth Mental Health Service Delivery in Kenya: Development and Usability Study
Source: JMIR Hum Factors. 2026 Jun 3;13:e79107. doi: 10.2196/79107 (PMC13276469; doi:10.2196/79107)
Supplement: Multimedia Appendix 3 [file humanfactors_v13i1e79107_app3.docx]

# Overview

This appendix provides a comprehensive technical specification of the shamiriOS architecture, including the Shamiri Digital Hub (SDH) for operational management and Rafi for youth-facing engagement. It covers the software stack, user access architecture, data model, security implementation, and technical feedback processes. For clinical workflow specification, see Appendix E (Shamiri Provider Platform).

# Software Architecture and Technical Stack

shamiriOS is built as a modular, multi-component platform with shared infrastructure but independent codebases and deployment models for each component.

## Shamiri Digital Hub (SDH): Operational Management Platform

**Technology Stack:** - **Backend:** Node.js with Express.js framework - **Frontend:** Next.js (React-based framework) hosted on Vercel - **Database:** PostgreSQL on Amazon RDS - **Analytics:** Metabase instance on Amazon EC2 - **Authentication:** JWT tokens with role-based access control (RBAC) at the API layer - **Deployment:** Cloud-hosted on Amazon Web Services (AWS)

### Version History:

**Version 1 (Late 2022 – Early 2023): No-Code MVP.** The initial prototype was built using Bubble, a no-code platform. This enabled rapid prototyping and early user feedback collection but reached scaling and customisation limits as operational requirements grew. Decision was made to migrate to a full-stack architecture.

**Version 2 (Early to Late 2023): Full-Stack Rebuild.** Complete rewrite in Node.js/Express backend and Next.js frontend. Introduced PostgreSQL for relational data management, JWT-based authentication with granular role-based access control, and RESTful API architecture. This version was deployed across five centralised Shamiri hubs by Q4 2023.

**Version 3 (Late 2023 – Present): Multi-Site and Analytics Integration.** Extended architecture to support multiple partner sites and schools with independent data isolation. Integrated Metabase analytics platform for real-time monitoring of operational and engagement metrics. Added offline-first data capture for scenarios with intermittent connectivity. Implemented automated daily database backups with point-in-time recovery capability.

## Rafi: Youth-Facing Engagement Platform

**Technology Stack:** - **Backend:** Node.js with Python micro-services for specific computational tasks - **Frontend:** Remix (React-based framework) with Vercel deployment - **Database:** PostgreSQL primary database; Digital Ocean Object Storage for file attachments (journal images, shared media) - **Mobile:** Native mobile apps (Android on Google Play Store; iOS on Apple App Store) - **Offline-First Architecture:** SQLite for on-device data storage; local-first data model with server synchronisation on reconnection - **Authentication:** JWT tokens with user-level authentication (role-based access was not required for youth-facing app)

**Development Approach:** Rafi development followed a co-design methodology with iterative feedback from youth users, peer counsellors, and implementation partners. Three major versions were released, each incorporating design refinements based on user research, usability testing, and deployment feedback.

## Technical Stack Comparison Table

| **Component** | **SDH** | **Rafi** |
| --- | --- | --- |
| **Backend** | Node.js, Express | Node.js, Python |
| **Frontend** | Next.js (web) | Remix (web) + React Native (mobile) |
| **Database** | PostgreSQL (AWS RDS) | PostgreSQL + DO Object Storage |
| **Authentication** | JWT + RBAC (5 roles) | JWT + user-level auth |
| **API Style** | RESTful | RESTful |
| **Offline Support** | Offline data capture (V3+) with auto-sync | Offline-first: core features fully offline |
| **Deployment** | Cloud-hosted (AWS); local server in development | Cloud-hosted (Vercel) + app stores (iOS/Android) |
| **Open Source** | Yes | Yes |
| **User Audience** | Staff/supervisors (role-differentiated) | Youth users (13–25 years) |

# SDH User Roles and Access Architecture

SDH implements granular role-based access control (RBAC) to ensure that users only access data and functions appropriate to their operational roles. Five primary user roles are defined:

| **Role** | **Primary Responsibilities** | **Data Access** | **Write Capabilities** |
| --- | --- | --- | --- |
| **Administrator** | System configuration, user account management, system-wide reporting | All data across all sites | All functions |
| **Hub Coordinator** | Day-to-day hub operations, attendance tracking, session scheduling, basic reporting | Hub-level data only | Attendance, sessions, basic assessments |
| **Clinical Supervisor** | Supervision of fellows, clinical case review, outcome monitoring, case escalation | Hub-level clinical and supervision data | Assessment scores, supervision notes, escalations |
| **Shamiri Fellow** | Direct service delivery, client assessment, session documentation | Client-level data only | Session notes, assessments, engagement records |
| **Read-Only Analyst** | Dashboard access, report generation, outcome evaluation | Aggregated data only (no identifiable information) | None; read-only |

Access control is enforced at the API layer: all requests include a JWT token carrying the user’s role, and the API validates that the requested resource falls within the user’s permitted scope before returning data or executing a write operation.

# Data Architecture and Security Model

## SDH Data Architecture

SDH is built on a relational database model with the following core entities:

- **Users:** Platform accounts with assigned roles and organisational affiliations
- **Organisations/Sites:** Shamiri implementation sites and partner schools
- **Programs:** Stepped-care programmes (e.g., “School-Based Mental Health Initiative”)
- **Sessions:** Individual client interactions (assessment sessions, group psychoeducation, individual counselling)
- **Attendance Records:** Tracking of client participation across sessions
- **Assessments:** Structured outcome measures (PHQ-9, GAD-7, functional impairment scales)
- **Reports:** Generated summaries of operational and clinical outcomes

All components communicate via RESTful APIs with JWT-based authentication and RBAC at the API layer, ensuring that data access is restricted to authorised users and that all cross-system communications are encrypted and audited.

## Rafi Data Architecture

Rafi employs an offline-first data model designed for low-bandwidth and intermittent-connectivity environments. User-generated data (mood check-ins, journal entries, goal tracking) is stored on-device using SQLite and synchronised to the server upon network reconnection. The architecture distinguishes between two categories of data:

**Data with local-first storage (fully functional offline):** - Self-guided content (affirmations, psychoeducational materials) - Mood check-ins and wellbeing tracking - Journal entries and reflections - Goal tracking and progress updates - User preferences and account settings

**Data with server-side authority (queued locally, synced on reconnection):** - Therapy session bookings and cancellations - Triage escalations and clinical routing decisions - AI-generated mood artwork - Community feed posts and interactions - Assessment scores for clinical review - Push notification receipt

Conflict resolution uses a **last-write-wins strategy** for user-generated content (mood check-ins, journals), allowing users to edit entries offline without complex conflict resolution. For clinical data (therapy bookings, triage escalations), **server-authoritative resolution** is employed: the server validates that a booking is still available or that an escalation is clinically appropriate before committing the transaction.

Sync failures are retried with exponential backoff, and the app provides users with clear feedback about whether their data has been saved locally (offline) or synchronised to the server.

## Security Model

**Data at Rest:** All data stored in PostgreSQL is encrypted using AES-256 encryption. Database credentials are managed through AWS Key Management Service (KMS) and stored in a secrets management system (1Password Teams), with access restricted to authorised engineers.

**Data in Transit:** All API communications between SDH, Rafi, and the Shamiri Provider Platform use TLS 1.2+ encryption. OAuth2 protocols govern API authentication and access scoping between systems.

**Backup and Recovery:** Database backups are automated daily with point-in-time recovery capability, enabling restoration to any point within a 30-day window.

**Audit Logging:** All access to clinical data is logged, including user identity, timestamp, action performed, and data accessed. Audit logs are retained for a minimum of 12 months.

**Data Minimisation:** The platform is designed for compliance with the Kenya Data Protection Act (2019) and incorporates data minimisation principles — collecting only the data fields required for operational workflows and outcome monitoring. PII (personally identifiable information) is separated from operational data and restricted to authorised clinical and administrative users.

# Structured User Feedback Process Across Development Versions

Across all three SDH versions, user feedback was solicited through structured processes integrated into development sprints.

## Version 1 Feedback (Late 2022 – Early 2023)

- **Format:** Weekly in-person feedback sessions with 3–5 Shamiri supervisors and hub coordinators
- **Methods:** Observational testing (watching users navigate the system), think-aloud protocols, and semi-structured interviews
- **Frequency:** Weekly during 8-week development cycle
- **Key Findings:** Usability issues with role-based access navigation; unclear session workflow; difficulty distinguishing assessment types

## Version 2 Feedback (2023)

- **Format:** Structured feedback at critical milestones plus rolling feedback channels
- **Methods:** Monthly usability testing sessions (n=5–8 users per session); monthly NPS surveys; open feedback Slack channel; helpdesk ticket analysis
- **Metrics Tracked:** System Usability Scale (SUS), Net Promoter Score (NPS), feature adoption rates, and error/support request patterns
- **Key Findings:** Strong satisfaction with core operational workflows (session scheduling, attendance tracking); requests for enhanced reporting and real-time data visibility; feedback on offline capability for low-connectivity sites

## Version 3 Feedback (Late 2023 – Present)

- **Format:** Continuous feedback channels plus structured quarterly deep-dives
- **Methods:** Monthly NPS surveys (n=20–30 respondents per round); helpdesk ticket thematic analysis; quarterly in-depth interviews (n=8–10); analytics dashboard monitoring of feature adoption; monthly check-in meetings with site coordinators
- **Metrics Tracked:** NPS scores, monthly active user rates, feature-specific adoption rates, session duration patterns, and support ticket resolution time
- **Key Findings:** Sustained high satisfaction scores (usability M=8.36/10, NPS M=8.63/100); operational burden reduction in attendance tracking and supervision workflows; desire for enhanced cross-site reporting and predictive analytics; feedback informing development of offline data capture for partner sites with poor connectivity.

# Deployment Outcome Data

## Implementation Evaluation Instruments

Platform performance was assessed using four measures:

1. **Usability:** Single-item satisfaction rating on a 1–10 scale: *"How easy is the platform to use?"* Administered to supervisors and hub coordinators post-deployment.
2. **Acceptability:** Net Promoter Score (NPS) item on a 1–10 scale: *"How likely are you to recommend this platform to a colleague?"* [adapted from Reichheld, 2003].
3. **Engagement:** Backend usage analytics tracking login frequency, weekly report submission rates, feature adoption by type, data entry completeness, and retention over time.
4. **Qualitative feedback:** Open-ended survey questions and semi-structured interviews conducted during and after training sessions.

These pragmatic measures were selected in preference to standardized implementation science instruments (e.g., System Usability Scale, Feasibility of Intervention Measure) due to the rapid, sprint-based development context, the simultaneous nature of development and evaluation, and field team requests for brief, low-burden feedback mechanisms. Future evaluations of the stable platform will employ standardized measures to enable cross-study comparison.

## Wave 1 Results: Centralized School-Based Hubs (Q4 2023)

Wave 1 targeted five centralized school-based implementation hubs across Nairobi and Kiambu counties, selected based on high program activity, digital readiness, and established supervisory infrastructure. Training reached 40 supervisors, 6 hub coordinators, and approximately 358 fellows, delivered through a combination of remote onboarding modules, in-person workshops, and follow-up helpdesk support.

**Table S1. Wave 1 SDH Deployment Outcomes**

| **Metric** | **Value** |
| --- | --- |
| Implementation hubs | 5 |
| Fellows (lay providers) trained | 358 |
| Supervisors trained | 40 |
| Hub coordinators trained | 6 |
| Students served | 20,697 |
| Survey respondents (supervisors + coordinators) | 22 |
| Usability rating (1–10) | M = 8.36 (SD = 1.49) |
| NPS / Acceptability rating (1–10) | M = 8.63 (SD = 1.46) |
| Weekly attendance reports submitted digitally | >90% of expected submissions |

Qualitative themes from post-deployment interviews: users emphasized the platform's ease of navigation relative to prior spreadsheet-based systems; real-time data capture was described as enabling supervisors to identify attendance gaps and respond within the same week rather than after manual data compilation; stipend workflow automation was the most frequently cited improvement, eliminating a previously manual two-to-three day reconciliation process. A minority of users reported intermittent difficulties with mobile data connectivity during data entry, reinforcing the priority of offline functionality for V3.

## Wave 2 Results: Decentralized Partner Sites (Early 2024)

Wave 2 expanded to six additional sites including peri-urban schools, community centers, and NGO-led decentralized hubs, bringing the total to 11 active sites. Site-level customization was introduced to accommodate diverse local workflows. Asynchronous training modules, onboarding videos, and quick-start guides were developed by user role; weekly virtual support check-ins were offered during each site's first month of operation; and site-level implementation champions were identified to support peer-to-peer learning.

**Table S2. Wave 2 SDH Deployment Outcomes (Cumulative to Q1 2024)**

| **Metric** | **Value** |
| --- | --- |
| Total active hubs | 11 |
| Total fellows supported | 1,195 |
| Total supervisors | 111 |
| Total students served | 76,344 |
| Site types | School-based (centralized), peri-urban schools, community centers, NGO-led partner hubs |

*Note: Disaggregated usability and NPS data by wave and site type are not available for the current reporting period. Data collection for a comparative evaluation is planned for 2025.*

## Rafi Deployment Metrics Across Cohorts

For comparative reference, the table below presents Rafi engagement metrics across all three deployment cohorts. See Multimedia Appendix 3 for full context.

**Table S3. Rafi Deployment Metrics by Cohort**

| **Metric** | **2022 Cohort (Rafi 1.0)** | **2023 Cohort (Rafi 2.0)** | **MKU 2024 (Rafi 3.0)** |
| --- | --- | --- | --- |
| Universities / sites | 7 universities | Multiple universities + student clubs | Mount Kenya University (MKU) |
| Students approached / enrolled | 1,349 registered; 857 enrolled | 1,008 registered | 5,143 students (full campus) |
| Session booking rate | 37.11% | 50.4% | Low (data pending) |
| Session attendance rate | 33.02% (of those booked) | 44.89% (of those booked) | Low (data pending) |
| Self-guided module engagement | 73.98% | 77.28% | ~10% sustained engagement |
| Key retention finding | Moderate engagement; need identified for personalization | Declining retention after onboarding | Significant drop-off post-sign-up; DAU decline |
