# Supplementary material for: Early Deployment of an Integrated Digital Platform (shamiriOS) for Scalable Youth Mental Health Service Delivery in Kenya: Development and Usability Study
Source: JMIR Hum Factors. 2026 Jun 3;13:e79107. doi: 10.2196/79107 (PMC13276469; doi:10.2196/79107)
Supplement: Multimedia Appendix 4 [file humanfactors_v13i1e79107_app4.docx]

# Overview

The Shamiri Provider Platform (SPP) is the clinical workflow component of shamiriOS, designed to support therapists in session documentation, treatment planning, and client outcomes tracking. Rather than building a clinical system from scratch, the approach was to adapt EasyClinic, an open-source electronic health record platform, and extend it with shamiriOS-specific features (therapy booking integration from Rafi, role-based supervision workflows, FHIR-aligned data model).

As of the time of manuscript submission, SDH and Rafi have been deployed and are generating operational and engagement data. The SPP reached production readiness after the observation period for this study. SPP deployment, clinical integration, and clinical outcomes will be reported in a subsequent publication. This appendix documents the architecture, design rationale, and roadmap.

# EasyClinic: Baseline Capabilities and Selection Rationale

## Why EasyClinic was selected

EasyClinic is a lightweight, open-source electronic health record (EHR) system designed for resource-constrained clinical settings. Selection criteria for the EHR baseline included: (1) open-source code availability; (2) low server infrastructure requirements; (3) design for low-literacy provider contexts; (4) existing mental health adaptability; (5) active maintenance; and (6) deployment experience in African healthcare systems.

EasyClinic met all criteria. It has been deployed in clinical settings across sub-Saharan Africa, has an active open-source community, and offers a lightweight data model and user interface suitable for task-shifted delivery contexts.

*Core features available in EasyClinic (out of the box)*

- Patient demographic registration and ID management
- Session scheduling and appointment tracking
- Clinical note documentation (free text and structured templates)
- Prescription management
- Outcome measurement with customisable assessment tools
- User role management and access control
- Report generation and basic analytics

# Adaptations for shamiriOS Integration

## Adaptation 1: Legacy Patient Record Migration

**Rationale:** The first cohorts of Rafi users had already generated engagement data (mood tracking, journal entries, therapy booking history). The SPP needed to inherit this historical data to avoid duplication and to provide therapists with complete patient context.

**Implementation:** An ETL (Extract, Transform, Load) process was built to migrate patient demographics, mood tracking history, and therapy booking records from Rafi’s PostgreSQL database to EasyClinic. PII stripping was applied to create de-identified research datasets while preserving identifiable data in the clinical system. Migration was completed by Q4 2023.

**Status:** Complete

## Adaptation 2: Real-Time Therapist Availability Integration

**Rationale:** Rafi’s therapy booking feature needs to know which therapists are available on which dates/times. Rather than requiring manual entry, the system should fetch real-time availability from EasyClinic.

**Implementation:** A bidirectional API was created between Rafi and EasyClinic. When a therapist creates or modifies their availability in EasyClinic, the Rafi app displays updated booking slots. When a client books through Rafi, the appointment is created in EasyClinic and the slot is locked against double-booking.

**Development approach:** API-first architecture with comprehensive error handling and fallback mechanisms (e.g., if the sync fails, users see a “contact us to book” option rather than a broken booking interface).

**Status:** Live and operational

## Adaptation 3: Subscription Plan Management via Rafi

**Rationale:** Clients using Rafi pay for therapy through a subscription or per-session model managed in Rafi’s backend. This subscription status must be visible to therapists in EasyClinic (to enable therapists to confirm payment before providing service) and must be updated in real-time as clients purchase or renew subscriptions.

**Implementation:** A subscription service API was created to sync subscription status from Rafi to EasyClinic. When a therapist opens a client record, they see current subscription status and session balance.

**Development approach:** Asynchronous event-driven synchronisation: when a subscription event occurs in Rafi (purchase, renewal, expiry), an event is published to an event queue and consumed by EasyClinic’s subscription service.

**Status:** In development

# FHIR Compliance Roadmap

## Rationale for FHIR Alignment

FHIR (Fast Healthcare Interoperability Resources) is an HL7 standard for structured exchange of health data. Alignment with FHIR offers several benefits: (1) potential for data exchange with other health information systems in Kenya’s health ecosystem; (2) support for longer-term interoperability as SPP scales; (3) alignment with global digital health infrastructure standards; (4) improved data portability if users wish to transition to other systems.

However, immediate full-schema migration to FHIR was assessed as high-risk, requiring system downtime, significant engineering investment, and introducing technical debt risk. Rather than a full migration, shamiriOS has adopted a phased API-layer approach: building FHIR-compliant API interfaces on top of the existing EasyClinic data model, with progressive schema migration as individual modules stabilise.

## Key FHIR Resources Mapped to SPP Workflows

| **FHIR Resource** | **SPP Function** | **Implementation Status** |
| --- | --- | --- |
| Patient | Client profiles: demographics, contact information, unified identifiers | Planned (Phase 1) |
| Practitioner | Therapist profiles: qualifications, specialties, availability | Planned (Phase 1) |
| Encounter | Clinical session records: type, date, participants, duration | Planned (Phase 1) |
| Appointment | Session scheduling: booking, cancellation, reminders | In progress |
| Observation | Assessment outputs: PHQ-9, GAD-7 and other screening scores | Planned (Phase 2) |
| Condition | Diagnoses and clinical conditions documented by therapists | Planned (Phase 2) |
| CarePlan | Treatment plans: goals, interventions, responsible practitioners | Planned (Phase 2) |
| ClinicalImpression | Therapist session impressions: preliminary diagnoses, symptom documentation, clinical insights | Planned (Phase 2) |
| Questionnaire / QuestionnaireResponse | Structured assessment instruments and patient responses (e.g., PHQ-9 as Questionnaire; scored responses as QuestionnaireResponse) | Planned (Phase 2) |
| RiskAssessment | Formalized risk evaluation: self-harm ideation, clinical escalation triggers | Planned (Phase 2) |
| DocumentReference | Links to clinical documents: session notes, treatment summaries | Planned (Phase 3) |
| EpisodeOfCare | Care episodes: sequences of encounters representing a clinical treatment period | Planned (Phase 3) |
| FamilyMemberHistory | Family mental health history as recorded by therapists | Planned (Phase 3) |
| Organization | Institutional structure: Shamiri entities, partner organisations, implementation sites | Planned (Phase 1) |
| Location | Physical and virtual session locations | Planned (Phase 1) |

## Phased Implementation Approach

### Phase 1 — Foundational Data Architecture

Priority actions: 1. Separate the Student/User entity (currently unified in Rafi) from the Patient entity in the clinical system, establishing a clean distinction between a platform user and a clinical patient 2. Introduce an Organisation table as the top-level entity to support multi-tenancy (multiple implementing organizations using the SPP within a shared infrastructure) 3. Separate clinical session records from operational session logs (currently merged in the SDH/Rafi schema) into distinct Encounter and Appointment records 4. Implement Patient, Practitioner, Appointment, Encounter, and Organization resources at the API layer

### Phase 2 — Clinical Data Enrichment

Priority actions: 1. Introduce Observation and Questionnaire/QuestionnaireResponse tables to support structured data entry for validated screening instruments 2. Implement ClinicalImpression and RiskAssessment resources to support structured therapist documentation 3. Introduce Condition and CarePlan to support formal diagnosis and treatment planning workflows

**Phase 3 — Documentation and Longitudinal Care**

Priority actions: 1. Implement DocumentReference for linking session note documents to patient records 2. Introduce EpisodeOfCare to enable longitudinal care episode tracking across multiple encounters 3. Implement FamilyMemberHistory to support comprehensive clinical intake documentation

# Security and Consent Architecture

The SPP security design reflects both general data security best practices and the specific requirements of mental health data in a research and clinical context:

- **Database credential management:** Production database credentials are managed via AWS Key Management Service (KMS) and stored in a secrets management system (1Password Teams), with access restricted to a defined set of authorised engineers. Credentials are rotated on a regular schedule and are not stored in application code repositories.
- **PII and downstream analytics:** Any downstream use of SPP data for analytics, AI model training, or research purposes requires explicit PII stripping through defined ETL (Extract, Transform, Load) processes before data is made available to non-clinical teams. This architecture ensures that research and AI teams access anonymised data only, without access to identifiable patient records.
- **Patient consent framework:** Patients are required to explicitly consent to two distinct purposes before their data is recorded: (1) storage and use for their own clinical care; (2) potential use in anonymised form for downstream analytics and research. Consent status is stored as a structured field in the patient record and gates data access for non-clinical purposes.
- **Inter-system communications:** All API communications between EasyClinic, Rafi, and SDH follow strict HTTPS encryption. OAuth2 protocols govern API authentication and access scoping between systems.

# Current Integration Status and Planned Next Steps

## Current state (at time of submission)

- Legacy patient record migration from Rafi and SDH: **Complete**
- Real-time therapist availability integration in Rafi booking interface: **Live**
- Subscription plan management via Rafi: **In development**
- Full deployment of SPP in clinical practice: **Integration testing phase**; formal deployment outcomes not reported in this paper

## Cross-Component Data Flows

[NEW] The three shamiriOS components exchange data through defined integration points: Rafi sends therapy booking requests and triage escalations to the SPP via API; the SPP shares session completion and clinical outcome data with SDH for programme monitoring; and SDH provides programme configuration data (active sites, assigned therapists, intervention protocols) to both Rafi and SPP. A full specification of cross-component data flows, including API contracts and data governance protocols, will be published alongside the planned SPP deployment evaluation.

### Planned next steps

- Complete Phase 1 FHIR data architecture implementation
- Full deployment of SPP across all active Shamiri clinical sites
- WhatsApp bot integration for session reminders and follow-up communications
- Web portal for client-facing access to session summaries and care plan information
- Deeper bidirectional synchronisation between SPP and Rafi for care plan visibility and in-app clinical communication features
- Phase 2 FHIR implementation (structured assessment data, ClinicalImpression, RiskAssessment)
